# Redesign and validation of a computer programming course using Inductive Teaching Method

**DOI:** 10.1371/journal.pone.0233716

**Published:** 2020-06-04

**Authors:** Iftikhar Ahmed Khan, Mehreen Iftikhar, Syed Sajid Hussain, Attiqa Rehman, Nosheen Gul, Waqas Jadoon, Babar Nazir

**Affiliations:** Department of Computer Science, COMSATS University Islamabad, Abbottabad, Pakistan; Universidad de Chile, CHILE

## Abstract

Inductive Teaching Method (ITM) promotes effective learning in technological education (Felder & Silverman, 1988). Students prefer ITM more as it makes the subject easily understandable (Goltermann, 2011). The ITM motivates the students to actively participate in class activities and therefore could be considered a better approach to teach computer programming. There has been little research on implementing ITM in computer science courses despite its potential to improve effective learning. In this research, an existing computer programming lab course is taught using a traditional Deductive Teaching Method (DTM). The course is redesigned and taught by adopting the ITM instead. Furthermore, a comprehensive plan has been devised to deliver the course content in computer labs. The course was evaluated in an experiment consisting of 81 undergraduate students. The students in the Experimental Group (EG) (N = 45) were taught using the redesigned ITM course, whereas the students in the Control Group (CG) (N = 36) were taught using the DTM course. The performance of both groups was compared in terms of the marks obtained by them. A pre-test conducted to compare pre-course mathematical and analytical abilities showed that CG was better in analytical reasoning with no significant differences in mathematical abilities. Three post-tests were used to evaluate the groups theoretical and practical competence in programming and showed EG improved performance with large, medium, and small effect sizes as compared to CG. The results of this research could help computer programming educators to implement inductive strategies that could improve the learning of the computer programming.

## Introduction

With the steady growth in automation and digitalization, there is a shortage of Computer Science (CS) graduates [1]. The Forbes Technology Council [2] identified 13 technology skills needed in the job market in 2018, and 11 of these skills directly or indirectly involve computer programming. This shows a greater employability potential of CS graduates. At the same time, ironically many CS graduates are unemployed [3]. One of the reasons for the unemployability of CS graduates (despite the potential shortage of skilled workers) could be that they are not able to learn critical programming skills. However, programming is an important course in the CS curricula, and it is a pre-requisite of most of the CS courses [4]. Since computer programming is a challenging logical task [5], programming courses are commonly regarded as difficult and often have the highest dropout rates [6]. Many researchers have studied the factors that cause difficulties for students in learning programming. Some of the reported factors are students’ lack of computing experience and lack of computing background [7]. Linden & Lederman [8] report that the students were unable to grasp memory-related concepts most of the times. Students were not able to create a clear mental model of memory allocations and de-allocations during program execution. Other causes of problems faced by the students were: (a) ineffective use of representation techniques for problem-solving (b) students’ inability of analyzing problems (c) inability to master the programming constructs and syntax and (d) ineffective use of teaching strategies for problem-solving and coding [9].

The Deductive Teaching Methodology (DTM) is predominantly used to teach programming at universities. Tarsoly & Valijärvi [10] define the DTM as a rule-driven, top-down teaching approach in which the teachers first introduce and explain the concepts relating to a subject matter and then encourage students to practically apply the concepts. Lecturing is the most prevalent approach in the DTM [11]. It encourages memorization and the students feel overwhelmed by it [10]. Also, it reduces curiosity, excitement and independent inquiry [10], which are required for creative work like programming. Since lecturing limits the students’ engagement to the learning material, therefore they are unable to develop a deeper understanding of the concepts [12]. As science and technology education requires the students to use the concepts towards problem-solving, and lecturing may not be suitable in this context [13]. Therefore, the DTM may not be an effective approach to teach an applied course like programming, and there is a research gap to explore alternative teaching methods.

The Inductive Teaching Method (ITM) is defined as an example-driven, bottom-up teaching approach in which the teacher provides students with a set of data and allows them to draw their own conclusions. The students observe that how the concept is used in the provided data, figure out the rule therefrom, and then verbalize it [10]. The ITM promotes effective learning in technological education as compared to the DTM [14]. Also, the students appreciate the ITM because it provides them with a better grasp of the course contents and makes it easier for them to understand the teacher [15]. The ITM motivates the students to actively follow the class activities, and therefore, it may be a better approach to teach programming.

The ITM has been used to teach CS courses like Software Engineering (SE) [16]. Also, the ITM is used to teach the Oracle tool to the students who already knew the basics of programming [17]. Both studies report promising results of the ITM. However, both studies assume that students should have basic programming skills. Since building basic programming skills itself is a challenge. Therefore, in this research, we aim to implement the ITM to teach the basic concepts of computer programming.

The teacher could afford little time in a semester system to select and organize the learning material by using an ITM approach. The additional challenge is to plan the execution of the contents as well as to deliver these contents [18]. Therefore, designing an ITM based course is highly challenging. In this research, we have designed the course contents for an introductory computer programming course based on an ITM approach. The learning content and the examples of a computer programming course used in the DTM courses are modified for the ITM course. In addition, a plan was chalked out for the teachers to deliver the designed course contents. The designed contents and the approach were evaluated by implementing the ITM course in an undergraduate computer science program at a university. Since considerable research showed the effectiveness of ITM, we have formulated the following hypotheses:

**H**_**1**_: Teaching a programming course using the ITM improves the students’ performance as compared to the traditional DTM course.

The existing literature on the ITM relates its effectiveness to increase student motivation and improve the perception of a course [18]. Since the ITM is a learner-centered teaching approach, the students may find it helpful. The students of a learner-centric algebra course were able to achieve the learning outcomes at a better rate. However, their perception of the course was not favorable [19]. So, student motivation of the ITM is an interesting research topic. Therefore, in this research, we also considered students’ perception of the ITM course. Thus, we formulated another hypothesis as follows:

**H**_**2**_: The student perceives inductive teaching approach easier to follow and comprehendible as compared to the deductive teaching approach.

The rest of the paper is organized as follows: Section 2 discusses the previous literature on the ITM. Section 3 discusses the process of designing the course contents of an introductory computer programming course using the ITM. Section 4 describes the experiment conducted to evaluate the effectiveness of the designed ITM course for computer programming, and section 5 presents the results of the experiment. Section 6 concludes the paper by summarizing the main findings, listing the limitations of this research, and pointing to some future directions.

### Literature review

This section presents a research background on ITM and its use in designing courses. The first sub-section describes the theoretical foundations of the ITM approach with an aim to underline the cognitive processes used by the ITM as compared to the DTM. The purpose of this discussion is to compare the two approaches using a theoretical lens and highlight their differences. The second sub-section describes the previous approaches of using ITM to teach CS subjects.

### Theoretical foundations of ITM

Traditional DTM uses lecturing to teach engineering and science. A lecture presents theoretical concepts followed by examples and practical activities to teach the application of these concepts. This way the students learn by applying general principles and theories to specific situations. Theoretically, this approach is based on *positivism*. According to this philosophical stance, engineering concepts exist as absolute knowledge and the purpose of a lecture is to let students absorb and consequently apply this knowledge.

The ITM is theoretically grounded in *constructivism*, which involves constructing general principles from specific examples [20]. The idea behind constructivism is that learners construct and reconstruct knowledge by using their experiences and by making sense of those experiences. The ITM presents new engineering knowledge as a continuation of prior knowledge and help in making new knowledge easy to relate and retain [20]. Further, since the ITM is based on a deep exploration of new knowledge, it may increase the motivation of students and discourage rote memorization with illogical thinking.

Another key difference between ITM and DTM is based on the roles of teacher and student in the learning process. In the DTM, the teacher is seen as an expert of a subject whose job is to deliver knowledge to the students, whereas the student is seen as a consumer of the knowledge. In the ITM, the students take an active role in learning and the teacher acts as a facilitator guiding the learning process towards success [21]. The active student participation can be achieved by restructuring the learning contents around questions and problems. The students are encouraged to collaborate for answering questions and solving problems to discover knowledge. The teacher can fill the gaps in the knowledge discovered by students using just-in-time teaching approach of the ITM [21].

Since the ITM is a learner-centric approach, it helps the students to learn creativity and problem-solving skills, which are essential for engineering disciplines [22]. Therefore, the ITM has a significant potential of success for teaching subjects in applied disciplines like CS. The following sub-section presents the ITM approaches to teach CS subjects.

### ITM in teaching CS subjects

According to Prince & Felder [20], the ITM is a broader term for many learner-centered approaches including:

- inquiry-based learning centered at answering open-ended questions,
- discovery learning involving self-directed discovery of knowledge,
- problem/project-based learning centered around solving problems and/or executing projects,
- case-based learning involving case studies and
- just-in-time teaching after exploratory assignments.

Some of these approaches are implemented by restructuring the learning design (like online learning, studio-based design workshops and student projects). Other approaches are implemented in a semester environment and a traditional classroom setting. The following paragraphs describe various approaches implementing the ITM in CS.

Köppe & Rodin [17] presented Guided Exploration (GE) as an inductive approach to teach tool-related concepts and techniques. The authors used it to design a course on the development of administration systems using Oracle APEX. The authors report that in general GE as instructional material was well perceived by the students. The learning objectives were achieved in a better way as compared to the old course. The results show that the ITM can be used to teach a tool, however, this cannot be generalized to teaching programming concepts. In teaching a tool, examples of the tool are either available via the help option of the tool or from the documentation of the tool. The student needs to follow the steps and understand the concepts. In addition, students with previous CS education found the GE more helpful [17].

Sedelmaier & Landes [16] used an inductive didactical approach to teach the concepts of SE. Particularly, they focused on the concepts like: the need of SE, software process models, requirement analysis and software design. The students were aware of Java programming language and of other CS concepts. However, this was their first exposure to the SE techniques. Jaime et al. [23] used a project-based approach to teach a project management course. The students developed four group-projects in a semester. These projects involved milestones and deliverables which are evaluated using peer assessment by group members. The authors reported a gain in the quality of the projects measured in the grades assigned by the instructor. This course was taught later in the curriculum, when the students were already familiar with the basics of SE. Teiniker et al. [24] used two inductive approaches to train the software engineers in software security, namely: a) an enquiry-based approach implemented to teach software vulnerabilities at code level and b) a project-based approach implemented to teach risk analysis at the level of software architecture. In the enquiry-based learning approach, the students were presented with snippets of source code containing software vulnerabilities. Additionally, automated attacks were implemented in the software repository. Firstly, the students performed code review and debugged the code to unearth the software vulnerabilities and to identify attacks. Secondly, the students fixed the identified software vulnerabilities and wrote test cases to verify the vulnerabilities’ successful removal. The authors also implemented a project-based learning approach, where the students were presented with application fragments and they were required to identify architectural risks in the fragments. Again, the students fixed the problems in the application fragments and tested them with penetration testing. The qualitative evaluation of both inductive learning approaches showed that the students found these approaches beneficial.

The learners involved in all the above narrated ITM approaches had prior knowledge of CS and programming. Since these learners were familiar with the computational thinking, they were able to explore the learning content presented as problems, examples, or projects. However, developing the motivation and interest of fresh CS students is essential to retain them in introductory as well as in advance CS courses [25]. Because the ITM may be helpful in motivating students [18], developing an ITM-based introductory course in CS is important. Dȩbiec [25] redesigned an introductory course to teach digital systems using the ITM. In this course, the author used demonstrations using Python interpreter followed by a lecture. The students took more interest in the lecture due to the demonstrations. The author evaluated the course qualitatively using student surveys and quantitively in terms of students’ attendance and grades. The results showed that students were motivated in the course and their attendance and grades improved.

The results reported by Dȩbiec [25] showed that the ITM can be successfully used to teach introductory courses in CS. Introduction to Computers & Programming (ITCP) is an important course in CS curriculum. Therefore, the aim of this research is to teach the introductory programming course to the students with little or no prior knowledge of CS. The next section describes the materials and methods used to accomplish the aim of the study.

### ITM course contents of ITCP

This section presents the proposed redesign of the DTM course of ITCP to an ITM course. Firstly, this section describes the redesign of the DTM course to an ITM course. Secondly, the ITM course contents are further described using an example ITM lecture and an ITM handout.

### Redesigning the DTM course to the ITM course

The contents of the programming course were already formulated by the National Curriculum Review Committee (NCRC) following the guidelines of the ACM/IEEE curriculum [26]. The title of the course was ITCP with two components namely: Introduction to Computers (ITC) and Introduction to Programming (ITP). The course consisted of 4 credit hours and 6 contact hours (3 contact hours theory and 3 contact hours of lab work per week). The existing DTM methodology t teach a computer programming course consisted of the three parts: 1) lecturing in the theory class describing the rules, 2) providing the examples and 3) practicing in computer lab. The DTM course contents described above are provided as S1 Appendix. The ITM course of ITCP was designed with the following three parts:

1. A handout for each lecture containing examples to be practiced by the students
2. Practicing the examples, observations, analysis, and extraction of rules by the students themselves without any help
3. Explanation of the rules by the teacher already observed or possibly overlooked by the students.

To clarify the difference between the two teaching methodologies, an example is provided below:

### Example ITM lecture

In the DTM course, students are introduced to the variable naming rules in the first part of a lecture, then some examples are provided for the rules in the second part of the lecture. In the third part of the lecture, students must practice the rules in computer lab. For example, students are taught that variable names in C/C++ programming languages cannot have space and special characters other than ‘*’ and ‘_’. To improve students' learning several valid and invalid examples are provided by following variables naming conventions. Some students note them down in their notebooks to practice later in the lab. The students are referred to textbooks for further details on variable naming rules.

**Fig 1:**
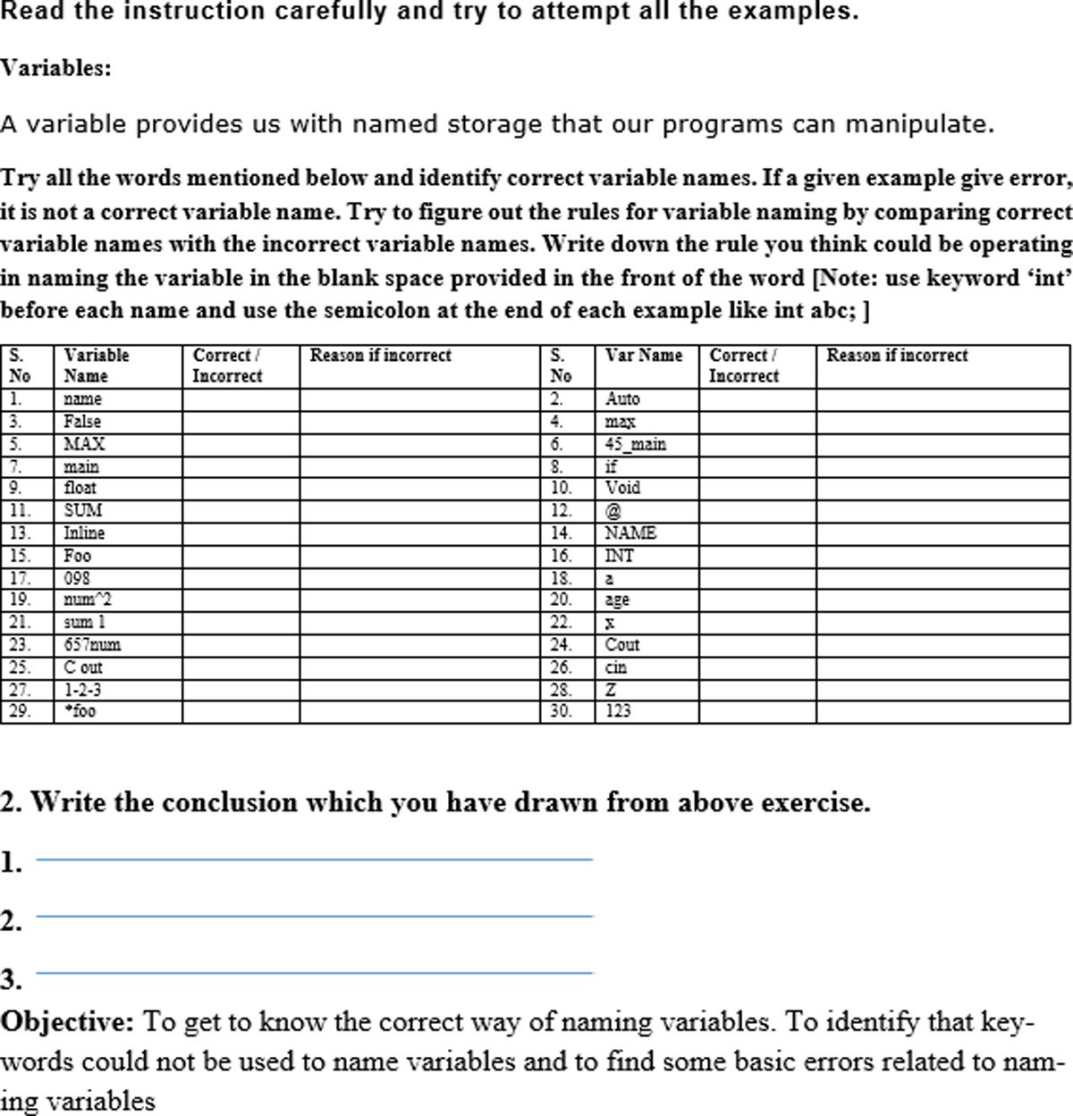
Example ITM handout. This handout contains variable names and corresponding spaces to write reasons if a variable name is incorrect. It also contains a conclusion section to write the discovered variable naming rules.

We changed this method of teaching using a discovery based ITM approach. Before learning to name variables correctly, a student must understand writing a correct programming statement. Therefore, in the lesson on variable naming, we first introduced the students with writing a correct programming statement. Then, we provided them with the prepared ITM handout (c.f. Fig 1) containing 30 variable names. The students were required to practice each of these variable names by using a variable declaration statement in an Integrated Development Environment (IDE). We intuitively included some variable names that were not following the correct naming convention to get failed compilation. The students were instructed to find out the reasons for the failed compilation of statements using the compilation errors as well as their intuition. They were required to write the reasons in the space provided against the statement in the handout (c.f. Fig 1). Further, they were required to write the variable naming rules in the conclusion section of the handout. A time of 50 minutes was allocated to practice the variable names. The last 20–25 minutes of the lecture were utilized by the teacher to discuss the handout’s examples on the board. The teacher wrote each variable name on the whiteboard and asked the class whether the variable name was correct. The students read their handouts and gave the reasons if a variable name was incorrect in their opinion. The teacher explained the right reasons for the incorrect variable names where necessary to fill the knowledge gaps in the variable naming rules discovered by the students. This process follows just-in-time teaching approach of the ITM [20].

Thus, the changes were introduced in the selection of the experiences (practices) and the organization of the learning experiences. Since the designed course contents were to be implemented in the university, we decided not to change the evaluation process and to keep it with the university schedule and methods as much as possible.

### Experimental setup

To test the effectiveness of the designed contents various studies were planned. The following sub-sections explain the study designs, the participants, methods, and data preparation.

### Study design

The design of the study was a quasi-experimental and a between-subject design. The demerits of the design as mentioned by Martyn [27] were considered and are recognized as follows. The allocation of the students to experimental or control group sections was not in authors control. Rather, the admission department of the university assigned the students to different sections as per their admission order. The early admissions are allocated to Section-A while late admissions to Section-B. The admission order, therefore, could also be a confounding factor. For example, early admission getters could be more motivated. Therefore, a pre-test was planned to accommodate the differences.

The decisions to deliver the designed ITM course to Section-A was made via the flip of the coin. This section will be termed as Experimental Group (EG) in the rest of this paper. The DTM course was used to teach the students of the Control Group (CG) of section B. As per the university rules, a section should have only a maximum of 45 regular students. However, repeaters could also join the sections, making the section size more than 45.

As the ITCP course was a 6-contact hour course, therefore, two different teachers were assigned to teach the EG and CG sections again on the flip of a coin. This action was taken to conform to the departmental rule that one teacher can teach a maximum of 9 contact hours. This factor is another potential confounding variable of a quasi-experimental design. To cope with the impact of this potential confounding variable, help from the administration was taken. The teachers with almost equal experience (6 and 7 years of teaching experience) were selected to teach the two groups. Furthermore, we have also considered the students' feedback of the teachers in the selection of the teachers, and each of them had 80–85% feedback in teaching the programming courses. In addition, assigning the EG and CG to two different teachers removed teachers’ potential bias towards the EG or CG.

This research involved teaching the course of ITCP to the EG using the proposed ITM based course contents/design and to the CG using the existing DTM based course contents. The study design, including the procedures of assigning the students to the EG and CG and assigning teachers to the groups, was discussed, and approved by the Departmental Ethics Committee–called as Project Research & Evaluation Committee (PREC). The students were informed about the study and they were asked to sign written consent forms. The procedure of taking consent and the consent forms were approved by the PREC. Only after signing the consent form, a student was included in the study. Further, the students had the option of dropping out of the course at any time of the course, but no student dropped out.

### Participants

The EG consisted of 53 students and the CG consisted of 42 students. The repeater students were also part of the class. They were either improving their grades or they failed the course previously and were retaking the course. The repeaters, therefore, were not included in the following analysis. There were 8 repeaters in the EG and 6 in the CG reducing the sample size to 45 in the EG and 36 in the CG. The mean age of the students was 19 years in both the groups.

### Methods

The course was taught as per the university schedule. In a week, two lectures each of 1 hour and 30 minutes and two consecutive labs each of 1 hour and 30 minutes with a 15-minute break were scheduled. The course contents corresponding to the programming language were delivered to the EG in computer labs only. The CG classes were conducted in the traditional way. ITC contents comprised 20% of the course and were introduced to the CG students in the first two weeks. The remaining ITP contents were taught in the rest of the lectures and computer labs. The theory was introduced in the class before the practical in the lab.

For the EG, ITC contents were introduced only in the theory classes and the ITP contents only in the labs. The brief contents of ITC were fully covered in the theory classes till 5^th^ weeks of the semester. Therefore, from the 6^th^ week, the ITP contents were taught in the theory classes as well as in the labs. However, it was ensured that every new concept is introduced in the lab using the ITM approach and the topic is further elaborated in the following theory class.

### Students’ performance measurement

The students’ performance in the ITM course was measured in terms of obtained marks. There were two term exams and a final exam in the semester. The post-test 1 was taken as 1st term exam and post-test 2 as a 2nd term exam. As per the university schedule, there was a requirement to conduct the exams of all academic departments in the same week. However, the exam department did not administer the term exams. Therefore, the CG and the EG were evaluated in the post-test 1 and post-test 2 with the same question paper. However, using the same question paper was not possible for the final exam because of the control mechanisms of the exam department. Moreover, we must eliminate the possible influence of teacher as a confounding variable on the students’ performance. Therefore, a programming competition (called post-test 3) was planned before the final exam. The conduct of post-test 3 also ensured comparison of the two groups independent of the teachers’ influence on the results. The summary of the tests planned to compare the two groups as per the university schedule is given in the Table 1 below.

**Table 1:** Summary of student exams. This table presents a summary of all the exams by describing the test name, type of test (theory, lab), the week of the semester in which the test was taken and the reason to conduct the test.

S. No	Test Name	Test Type	Week of the semester	Reason
1	Pre-test	--	1^st^	This test was planned to assess the logical and mathematical competency level of the students of the two groups before the actual experiment. It acted as a baseline to find differences between the two groups if there are any.
2	Post-test 1	1^st^ term theory	6^th^	The 1^st^ term and 2^nd^ term theory exams were taken in the written form to probe a student’s conceptual understanding of the programming skills.
3	Post-test 2	2^nd^ term theory	11^th^	
4	Post-test 1	1^st^ term lab	6^th^	The 1^st^ term and 2^nd^ term lab exams, consisting of computer programming tasks were taken to measure the students’ programming and problem-solving skills.
5	Post-test 2	2^nd^ term lab	11^th^	
6	Post-test 3	Lab	15^th^	This exam was conducted to evaluate the students’ performance after eliminating out the confounding variable like the influence of the teacher.

The remaining part of this section describes the procedures of the tests listed in Table 1.

#### Pre-test

We conducted the pre-test to establish a baseline of the students’ competency level in basic mathematics and analytical logic. It was important to have the baseline competency level for each student so that we could measure the differences of the DTM course and the ITM course in developing the theoretical knowledge and programming skills of the students.

Since the DTM and the ITM courses were offered to the students of first semester, we could potentially use the results of the university entrance exam as a baseline. However, these results were not available to us at the time of the experiment because of the students’ privacy. Hence, we had to prepare another test comprising of 30 questions, where 15 questions were from basic mathematics and 15 questions were from analytical reasoning.

A set of instructions were given to the students before starting the test. The students were prohibited to communicate with each other, to erase, to cut, to overwrite or, to shade multiple circles against answers to a question. It was communicated that doing so will result in zero marks for that question.

#### Post-test 1 (theory and lab)

Since CG followed the traditional approach, therefore, students in this group were introduced to the theory first and then practical implementation in the lab. However, for EG, the lab teaching plan was changed. In the first lab, basic concepts about a programming language were given. A metaphor example of natural languages was equated with that of the computer programming concepts, which highlighted the importance of computer programing. They were given a clear understanding of the tools required throughout the course for the practical part of the subject. Several basic examples were given to the students that allowed them to build familiarization with the IDE.

As discussed earlier, each lab for EG was divided into three parts. The first part consisted of 15 minutes, where the teacher presented examples of the planned lecture topics. The second part consisted of 50 minutes, in which handouts (discussed in designing the curriculum part) were given to the students. The students were required to follow the instructions given in the handouts and complete the provided tasks using their computers. They were required to fill in the blank spaces after observing the results of the practical work they completed. Finally, the third part reserved 20–25 minutes for discussion on the given topics. The teacher was required to discuss the results of the handouts, highlighting the relevant rules, and any other implications relevant to topics.

After 5 weeks of the course progress, post-test 1 (theory and lab) was conducted in the sixth week. The tests were prepared by the teachers of both groups collaboratively. There were three parts in the post-test 1 theory. Part A consisted of multiple-choice questions, part B consisted of programming problems having short answers and part C consisted of some lengthy programming problems. All the topics taught in the labs were included in the test. However, ITC part taught to EG and CG was not a part of the exam. Both the teachers agreed to cover the ITC part in the assignments and quizzes only. The lab post-test 1 was conducted in a two-hour lab session.

#### Post-test 2 (theory and lab)

The post-test 2 (theory and lab) was conducted in the 11^th^ week. The contents of the Post-test 2 included all the programming related contents that were used to examine students in Post-test 1. In addition, new contents taught during the 7^th^ week to 11^th^ week were also included in the Post-test 2. Like Post-test 1, Post-test 2 also consisted of a theory part and a lab part. Similarly, the format of the post-test 2 (theory and lab) exams was like the format of the post-test 1 (theory and lab) exams.

#### Post-test 3

The DTM course and the ITM course were taught by different teachers. A uniform evaluation of the students in Post-test 1 and Post-test 2 was carefully planned by setting the same question papers for the students of the DTM course and the ITM course. Nonetheless, the effect of teachers was a confounding factor for the student evaluations. To account for the effect of the teachers, we have decided to conduct Post-test 3, which was a programming competition evaluated by a third teacher. We selected an equal number of regular students from both the groups. We selected 6 students with high scores, 6 students with scores nearest to lab average scores and 6 lowest scorers from both the EG and CG for the test. The total sample size was 36, and the test was designed by the teachers of both the groups collaboratively. Three programs of simple, average, and high complexity were part of the test. It was made sure that each successful program should have at least five statements including declaring variables and implementing logic. The test question paper was re-checked for ambiguities and errors by another teacher (not involved in teaching the two groups). Students were allocated one hour to complete the test. It was announced that the best programmer will be awarded a gift of 8GB USB drive. On the test day, 33 out of 36 students participated in the test. This included 17 students from EG and 16 students from CG. We requested an independent reviewer, who was not involved in the study, to evaluate the results. He was provided with the following marking criteria:

If the program compiles successfully with no errors and has at least five statements then allocate 7 marks, if logic is correct resulting into a correct output then allocate 10 marks, and if only the output is correct then allocate 3 marks. Each program was of worth 20 marks. The evaluator could give any marks between the minimum and maximum marks deciding on the accuracy of the results. The results from the peer were averaged to get final marks and their percentage out of 100 was calculated.

## Analyses and results

The previous section discussed the procedures of four tests conducted to evaluate the students’ performance (c.f. Table 1). This section describes the statistical analysis on the students’ marks acquired in these tests and the results obtained from the analyses (c.f. subsections pre-test 1, post-test 1, post-test 2 and post-test 3). The general implications of the ITM on the students’ performance are discussed in the subsection “Implications of the ITM on students’ performance”. The second hypothesis relates to the effect of the ITM and the DTM on the students’ perception. Therefore, we have conducted a survey using a questionnaire as described in the subsection “Students’ perceptions about the ITM”.

As per design, the first appropriate analysis considered was a Mixed Repeated Measure ANVOA (MRMA). Therefore, a MRMA was conducted by taking Pre-test, Post-test 1, Post-test 2, and Post-test 3 marks converted into percentages as within subject variables and Groups (EG, CG) as between subject factors. Due to significant Mauchly's test of sphericity, Greenhouse-Geisser repeated measure ANOVA model was considered. Though the ANOVA model appeared to be significant (*F* (3, 23) = 12.75, *p* < 0.01), but between subject main effect computed to measure differences between EG and CG was not significant (F (1, 25) = 0.014, p = 0.9) with an effect size showing 10% of variance of the model can be explained by the group differences. Therefore, as per recommendation by Brambor, Clark and Golder [28], we conducted the simple effect analysis in the form of ANOVAS’ and ANCOVAS’. Following sub-sections describe analyses for each of the test conducted.

### Pre-test

The analysis was conducted to find the differences between the logical and mathematical competencies of the two groups. The students’ correct answers in the analytical and the mathematical part were recorded separately, converted into a percentage out of 100 and were added to get total marks. A one-way ANOVA was conducted by taking marks of the mathematical, analytical as well as the sum of both as dependent variables separately. The groups (EG, CG) were taken as a fixed factor. The groups, EG and CG also represent a confounding variable in the admission order and therefore was not analyzed separately via an ANCOVA.

**Fig 2:**
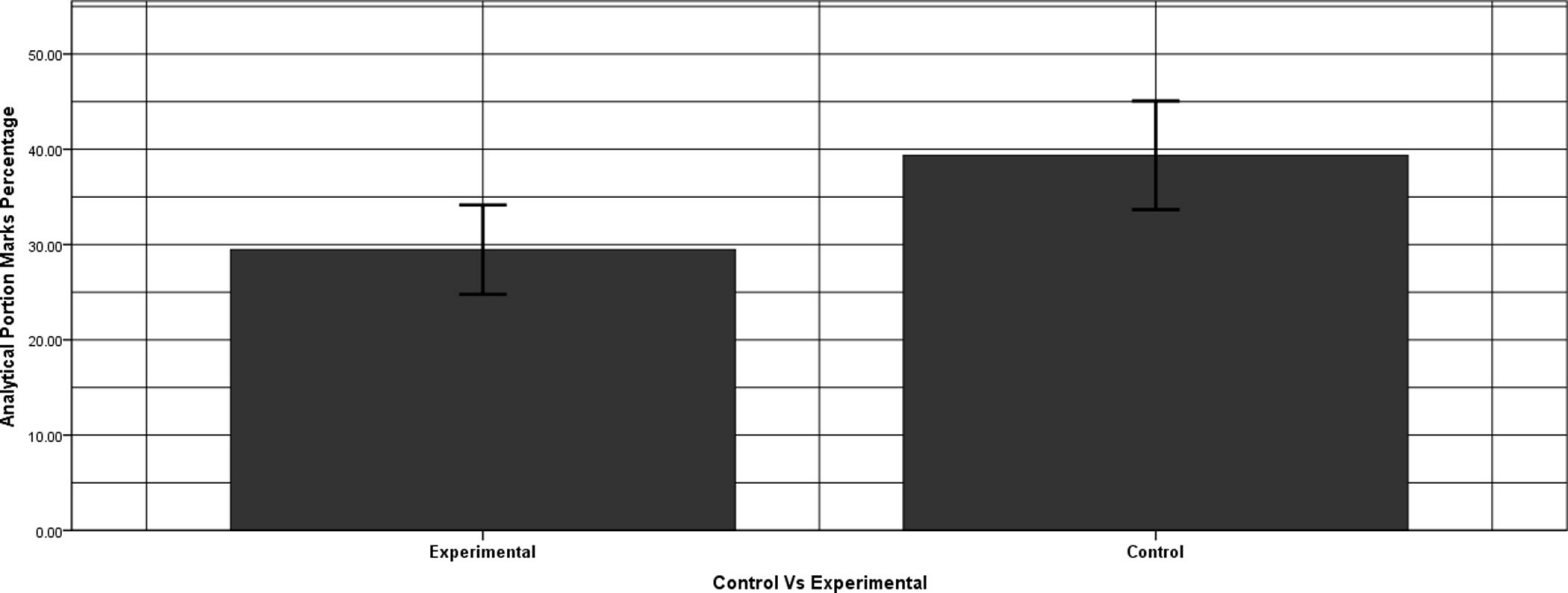
Marks acquired in the analytical portion of the pre-test. This bar chart shows the marks acquired by EG and CG in the analytical portion of the pre-test with 95% CI.

The results revealed no significant differences between the groups’ total marks and no significant differences between groups mathematical part. However, the groups differed significantly in their analytical competency level as CG being better in the analytical skills (*F* (2, 58) = 9.34, *p* = .003) with (*μ* = 39.38%) as compared to EG with (*μ* = 27.37%). The effect size (η² = 0.12) had a large effect as per Cohen [29], who demonstrated that η² of 0.01 is small, 0.059 is medium and 0.138 is a large effect size (c.f. Fig 2).

### Post-test 1

A one-way ANOVA was conducted considering post-test 1 theory and lab marks percentages as dependent variables and groups (EG, CG) as fixed factors. In addition, attendance of the students was not a controllable factor over 5 weeks of the study. Therefore, ANCOVAs was also conducted by taking post-test 1 theory and lab marks percentages as dependent variables, the groups (CG, EG) as fixed factors, and theory and lab attendance converted into percentages as a covariate.

**Fig 3:**
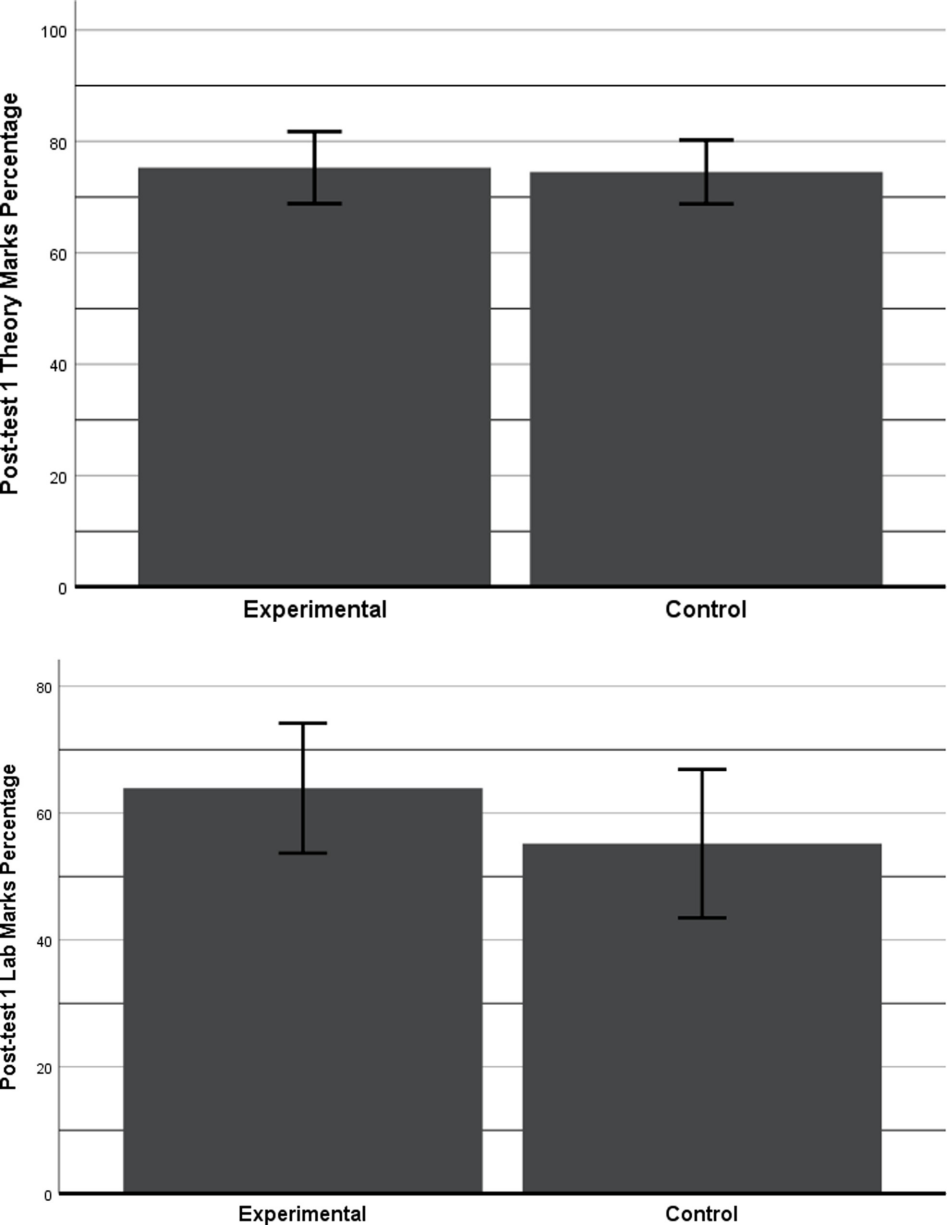
Results of the post-test 1 (theory and lab). a) This bar chart shows the adjusted marks in percentage of post-test 1 (theory) with 95% CI. b) This bar chart shows the adjusted marks in percentage of post-test 1 (lab) with 95% CI.

The results of theory marks showed no significant differences between the two groups with a simple ANOVA analysis. However, when attendance was considered as a covariate, the results revealed a significant difference between the means of the two groups *F* (2, 73) = 6.60, *p* = 0.012. The results show EG having better-adjusted marks (adj *M =* 74) as compared to CG (adj *M = 72*.*9)* with an effect size (η² = 0.08) which falls between medium and large as per Cohens’ distribution (c.f. Fig 3A).

The results of the lab marks also showed no significant differences between the two groups with an ANOVA analysis. However, when lab attendance was considered as a covariate, the results revealed a significant difference between the means of the two groups F (2, 73) = 3.96, p = 0.05. The EG showed improved adjusted marks (adj M = 64.3) as compared to CG (adj M = 55.9) with a medium effect size (η² = 0.05) as per Cohens’ distribution (c.f. Fig 3B).

An ANCOVA was also conducted by taking theory and lab marks percentage as dependent variables, groups (EG, CG) as fixed factors and percentage of pre-test analytical marks as covariate. The results showed no significant differences even after adjusting analytical marks differences.

### Post-test 2

A one-way ANOVA was conducted considering post-test 2 theory and lab marks percentages as dependent variables and groups (EG, CG) as fixed factors. Furthermore, ANCOVAs was conducted by taking post-test 2 theory and lab marks percentages as dependent variables, the groups (CG, EG) as fixed factors, and theory and lab attendance converted into percentages as a covariate.

**Fig 4:**
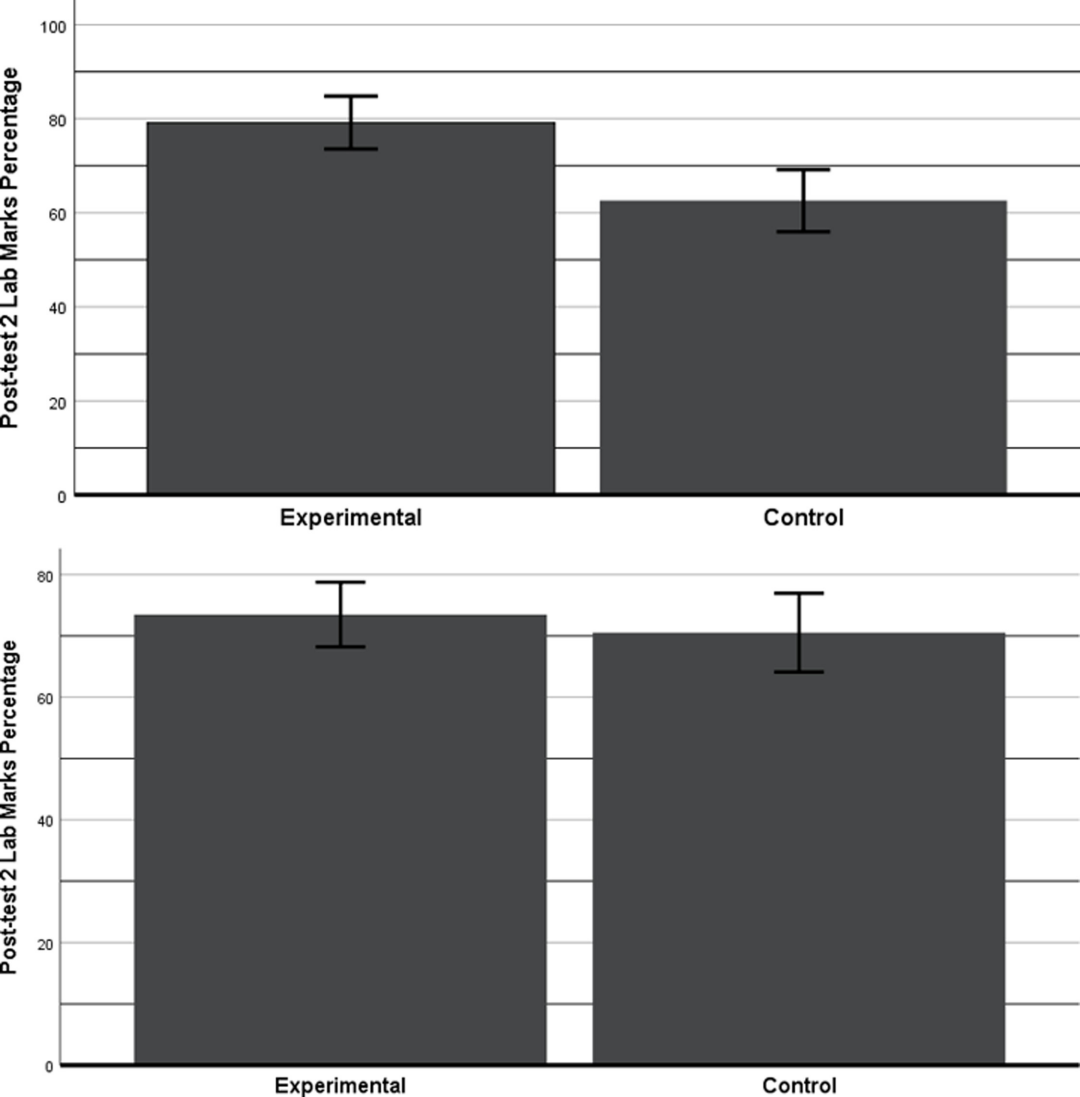
Results of the post-test 2 (lab). a) This bar chart shows the marks in percentage of post-test 2 (lab) with 95% CI. b) This bar chart shows the adjusted marks in percentage of post-test 2 (lab) with 95% CI.

The ANOVA showed no significant differences between the two groups theory marks. An ANCOVA conducted on theory marks again revealed no significant difference between the groups. However, ANOVA showed a significant difference in the lab marks (*F* (2, 72) = 14.6, *p* = 0.0003) with a very large effect size (*η²* = 0.16) in EG favor. The mean marks of EG (*μ* = 79.19%) were better than CG (*μ* = 62.58%) (c.f. Fig 4A). An ANCOVA also showed a significant difference between the groups’ lab marks (*F* (2, 70) = 3.9, *p* = 0.05). The EG showed improved adjusted marks (adj M = 73.47%) as compared to CG (adj M = 70.51%) with a medium effect size (η² = 0.05) as per Cohens’ distribution (c.f. Fig 4B).

An ANCOVA was again conducted to test the effect of pre-test analytical marks on the course theory and lab marks. Theory and lab marks percentage was taken as dependent variables, groups (EG, CG) as fixed factors and percentage of pre-test analytical marks as covariate. The results showed no significant differences between groups theory marks. However, there were significant differences between the groups’ lab marks (*F* (2, 64) = 7.66, *p* = 0.001). The EG showed improved adjusted marks (adj M = 80.14%) as compared to CG (adj M = 61.81%)

### Post-test 3

A one-way ANOVA was conducted considering post-test 3 marks percentages as dependent variables and groups (EG, CG) as fixed factors. Furthermore, ANCOVAs was conducted by taking post-test 3 marks percentages as dependent variables, the groups (CG, EG) as fixed factors, and an average of the students’ lab attendance in percentage up to week 10 as a covariate.

**Fig 5:**
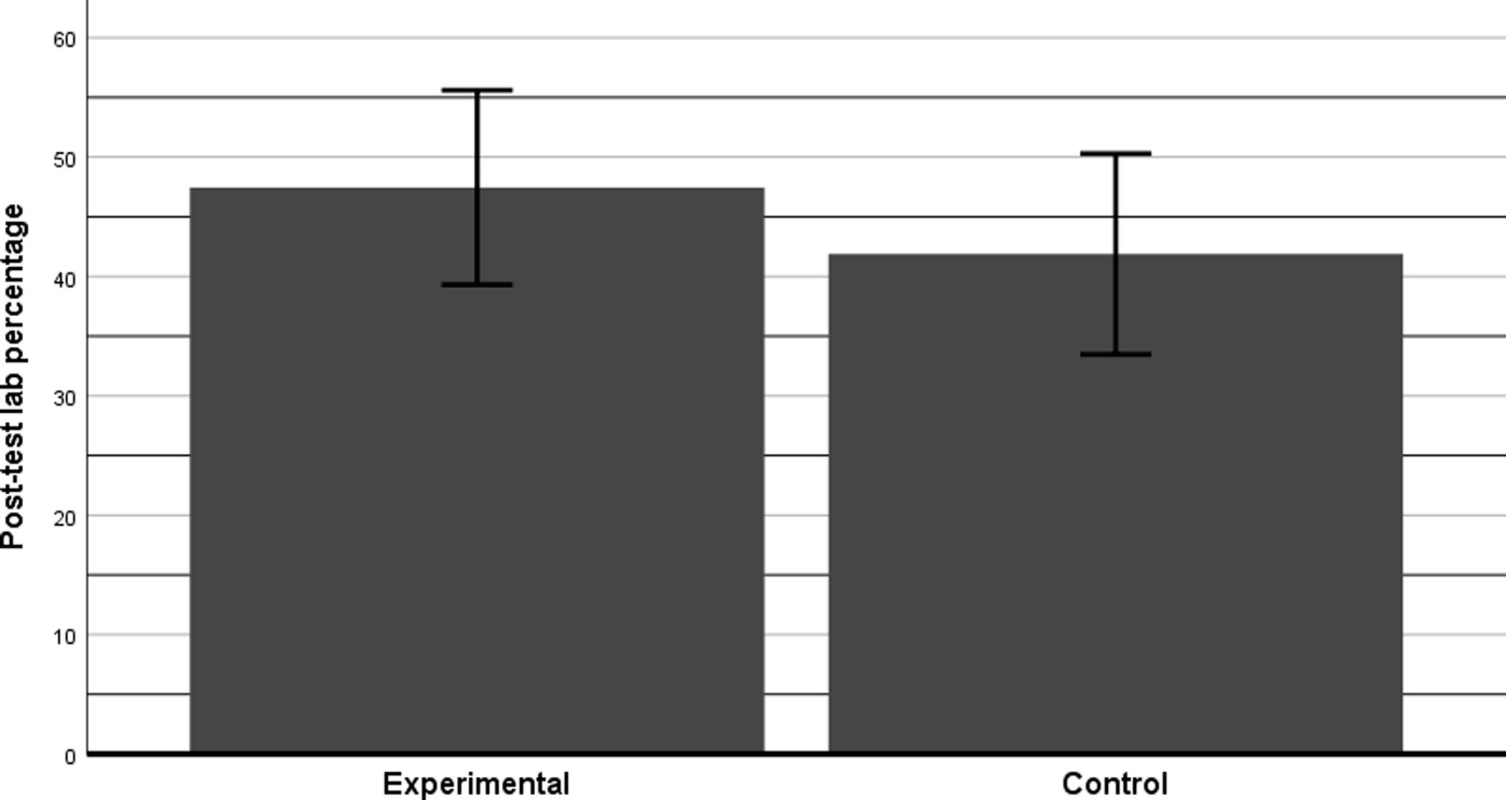
Adjusted marks in percentage of post-test 3 with 95% CI.

The results of post-test 3 showed no significant differences between the two groups even after adjusting for attendance. However, the mean adjusted score of EG was 47.45% and that of CG was 41.87% having a 5.58% difference of marks with an effect size between small and medium (c.f. Fig 5). According to researchers like [30], in education research, a difference of 5 or above (1 Grade) could be categorized as “quite substantial”. This is also evident from the effect size η² which is between small and medium effect sizes. In addition, no significance could also be attributed to the small sample size. However small sample size was not in the control of the authors due to the design of the study and availability of the lab space. The lab space could accommodate only 35–40 students.

Furthermore, an ANCOVA was conducted by taking theory and lab marks percentage as dependent variables, groups (EG, CG) as fixed factors and percentage of pre-test analytical marks as covariate. The results showed no significant differences even after adjusting analytical marks differences.

### Implications of the ITM on students’ performance

**Fig 6:**
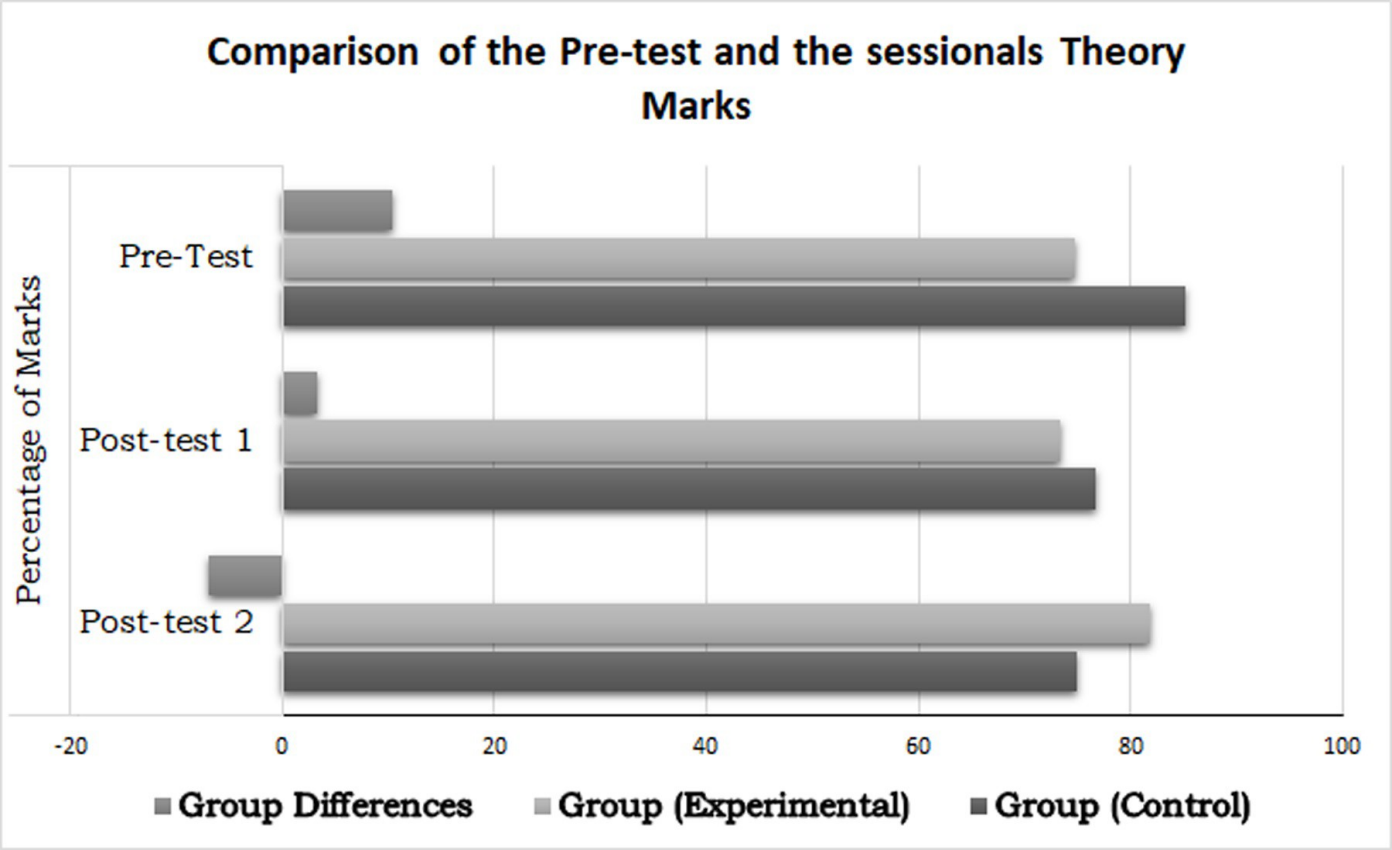
Results of pre-test, post-test 1 (theory) and post-test 2 (theory) without repeaters data.

**Fig 7:**
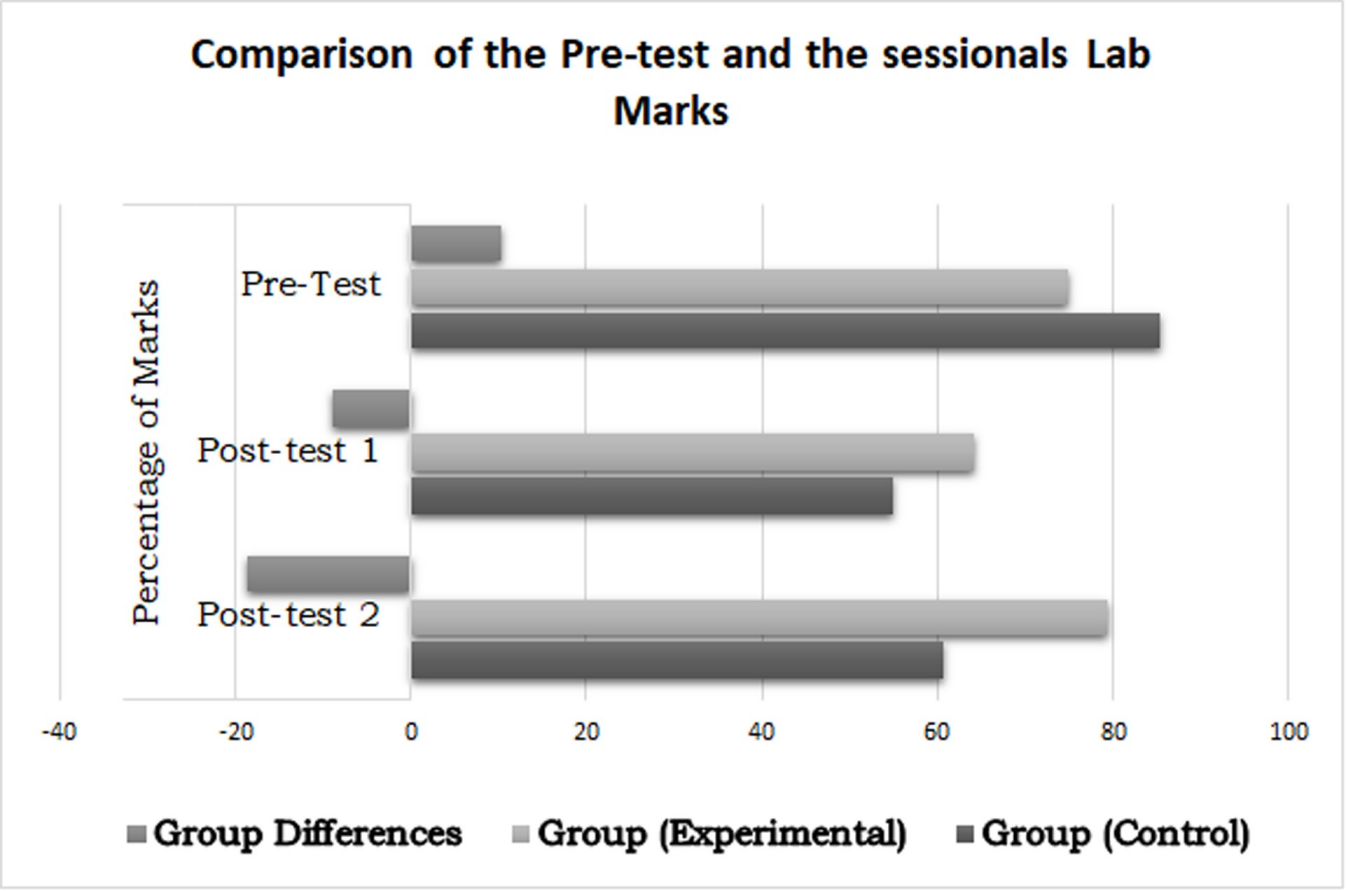
Results of pre-test, post-test 1 (lab) and post-test 2 (lab) without repeaters data.

A pre-test showed no significant differences between the groups. However, the portions (Analytical, Mathematical) analysis showed significant differences between the groups in analytical portion with EG students less fluent in the analytical portion. As analytical reasoning is closely related to programming capabilities, therefore, it could be concluded that EG programming like abilities were lesser than the CG. However, after introducing the EG to ITM, the analytical abilities were observed to be improving along with the time as depicted by the decreasing group differences visualized in Fig 6 below:

Fig 7 shows the differences between the groups’ pre-test score, 1^st^ term and 2^nd^ term lab scores and post-test 3 scores (considered as a lab because of its close resemblance to the lab exam). As is clear from Fig 7, the control group pre-test scores were better. In the 1^st^ term, the experimental group improved, and in 2^nd^ term it showed significantly better performance as compared to the control group. Although EG did not show any significant improvement in post-test 3, yet their improvement was between small to medium effect size. The inductive method application to teach the course is possibly a major contributing factor.

### Students’ perceptions about the ITM

To test the hypothesis H_2_, we collected data from the EG students about their perception of the course. In the past, all the student in EG completed their degrees which were based on DTM. Therefore, their perception of the course taught by a new method was considered a crucial factor. To find out students' perception related to the ITM, we conducted a qualitative analysis. A questionnaire was designed to get information about students’ perception of ITM. There were five questions in the questionnaire as shown in Table 2. The answering choices were: ‘strongly agree’, ‘agree’, ‘neutral’, ‘disagree’ and ‘strongly disagree’ ranging from 5 to 1. The questionnaire was distributed among the students after the final exam to ensure the feedback from all the potential students. Answering the questionnaire was optional. Out of the 45 regular students, 42 students submitted their feedback. The data was stored in an excel file and exported to SPSS for analysis.

**Table 2:** Survey questions.

Q. Id	Questions	Likert Scale Options
1	Do you agree that the teaching method (ITM) employed in this course clarifies more as compared to methods employed earlier to teach you?	1. Strongly disagree --
		5. Strongly agree
2	Do you feel that ITM should be implemented in other subjects?	1. Strongly disagree --
		5. Strongly agree
3	There is a difference between your earlier 12 years of learning and the learning of lab component in this course?	1. Strongly disagree --
		5. Strongly agree
4	Do you realize that programming is important in software engineering?	1. Strongly disagree --
		5. Strongly agree
5	Does the teaching method help you to actively participate in the class?	1. Strongly disagree --
		5. Strongly agree

#### Internal consistency of survey

An internal consistency analysis was conducted on the ITM satisfaction survey (c.f. Table 2) comprising 5 items. Cronbach’s alpha showed reliability alpha value equals to 0.65. Taber (2018) in his review cited studies related to science education that considered alpha level above 0.6 as acceptable. Various other studies and statisticians consider an alpha value of above 0.7 as acceptable (Taber, 2018). A further analysis revealed that the most items are worthy to be retained in the scale as the alpha decreases if any of them deleted from the scale. The exception is item 4 (Q4), whose deletion would increase the alpha to 0.67 which still less than 0.7. This item (asking about importance of programming) seems to be unrelated to the other questions which are all asking about ITM methods. Due to closeness to the acceptable alpha value, a further detailed analysis is conducted as explain below in the “Results” sub-section.

#### Results

**Fig 8:**
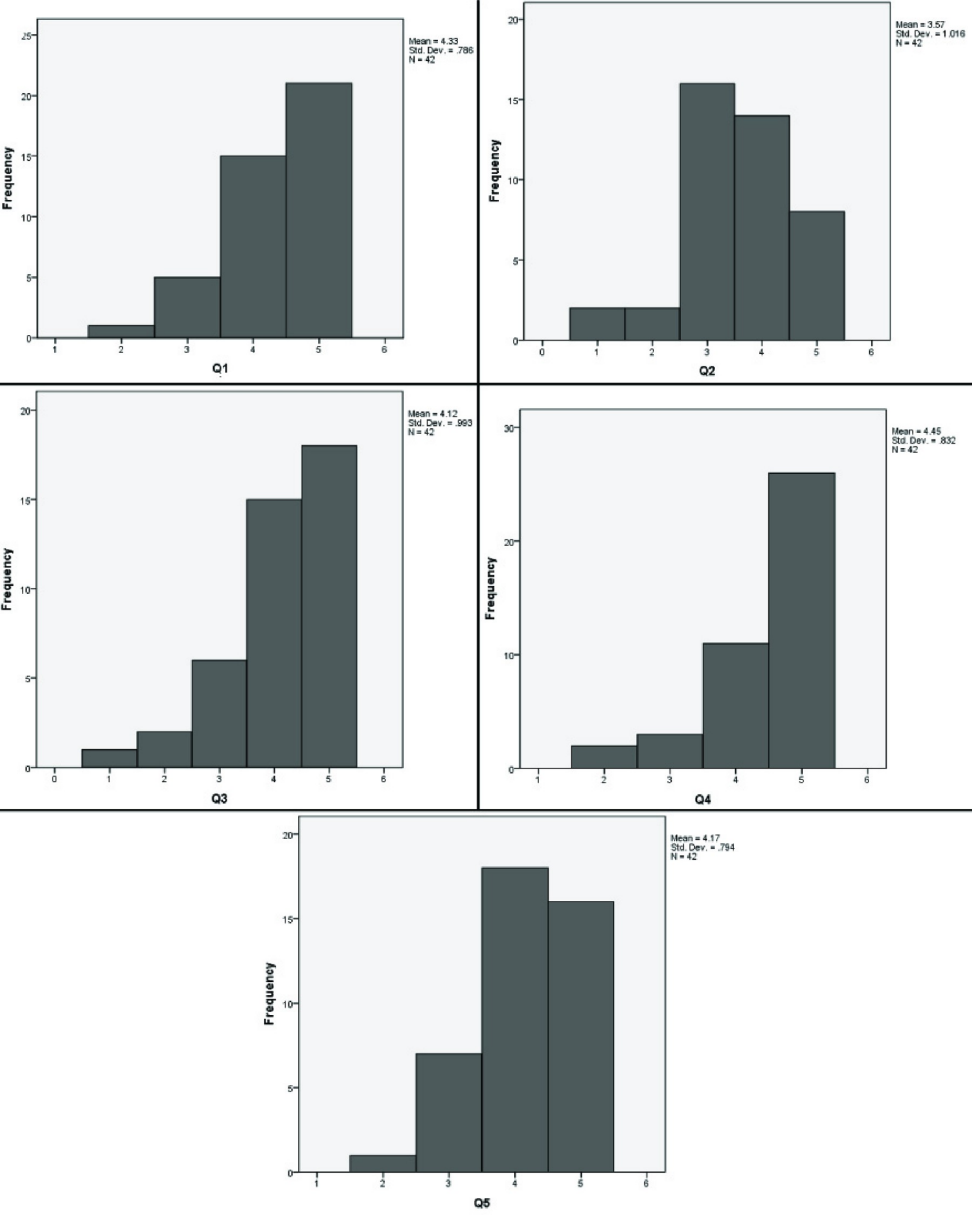
Distributions of response against Q1-Q5 as histograms.

A parametric t-test, as well as non-parametric one sample Wilcoxon’s signed rank test, were conducted by using the data from all the questions given in Table 2 as test variables. The test value was chosen to be 2.5 the mean value of the Likert scale. Student satisfied with a variable would have the value over 2.5, whereas unsatisfied students would rank the value below 2.5. The results indicated that the students strongly agreed that ITM clarifies programming (Q1). They believed that the inductive methodology should be implemented in other subjects as well (Q2). A significant number of students think that the course they learned via ITM was different from their earlier 12 years of education (Q3). This methodology also helped the students to understand the importance of programming in SE (Q4) as well as helped to actively participate in the class (Q5). The overall outcome of the questionnaire resulted in the acceptance of our hypothesis H_2_, which states that students perceive a course taught via ITM easier to follow as compared to the course taught via traditional teaching method (DTM). The distribution of the answers as histograms with means and standard deviations are illustrated in Fig 8 below. The N = 42, and p < 0.01 is constant for all the distributions.

## Conclusions and discussion

This research was conducted to study the implementation of an Inductive Teaching Method (ITM) course on computer programing. For this purpose, we have designed the course contents of an introductory course in computer programing at the undergraduate level. The effectiveness of the designed ITM course was validated by implementing it in a regular semester and comparing it with a Deductive Teaching Method (DTM) course using an Experimental Group (EG) and a Control Group (CG) respectively. The students’ performance was measured in terms of their marks (H_1_) and students’ perception of the ITM course (H_2_). To test H_1_, the students’ performance was evaluated using the pre-test, the post-test 1, the post-test 2 and the post-test 3. The analytical portion of the pre-test revealed that the CG was significantly better in analytical reasoning as compared to the EG. However, these differences appeared to be reduced in the groups in the subsequent evaluations (post-test 1 and post-test 2). To test the H_2_, a questionnaire was designed to collect subjective data on the students’ perception of the ITM and DTM. The results indicate that the students agreed that the ITM clarifies the programming concepts. They perceived that the ITM course also helped them to realize the importance of programming in Software Engineering (SE) and it should be implemented in other subjects as well. Moreover, a significant number of students believe that the ITM course was different in terms of learning and allowed active class participation as compared to their earlier 12-years learning using the DTM.

The improvement in the students’ performance when using ITM is aligned with the related literature. For example, Sedelmaier and Landes [16] found that the students had a better grasp of the SE concepts when they were taught using the ITM. Similarly, Koppe and Rodin (2013) reported promising results of the ITM. They found an increase in understanding of the Oracle tool when taught via the ITM. Other studies have also found promising results of the ITM but all of them assume that the students should have Computer Science (CS) knowledge and programming skills. The results of this research showed with small to medium effect size that the ITM can also improve the performance of the students having no prior knowledge of CS.

The previous research on the ITM has reported contradictory results on students’ perception of the ITM courses. For example, the DTM was found to improve students’ perception and student motivation [18; 25]. However, studies like Van Sickle [19] found that the students were less motivated in an ITM course. The results reported in this research showed better students’ perception of the ITM course as compared to their previous experience of the DTM.

This research implies that the educational institutions could introduce the ITM in the SE and CS departments. However, before implementing this approach on students with no prior knowledge of CS, more research is needed with bigger sample sizes and by eliminating possible confounding factors. This research could be considered as a foundation for the purpose as this research introduced the designed contents and a plan which could be further tested in another part of the world as well. The limitations like change in computer lab time (90 minutes) could invalidate the findings. The designed course contents for ITM can be further refined. Missing constructs not included in the designed could be included. For example, there could be a new lecture on the use of logical and combination of logical operators. The lectures on looping and control can be enhanced by dividing them into syntax understanding and problem-solving parts.

Some more limitations include a between-subject quasi-experimental design. There were some inherent flaws of a between-subject design in the study. For example, the teachers and the classroom environment were different for the EG and the CG. Random allocation of the students to the EG and the CG was not controllable. Future studies may be conducted to accommodate these factors. Furthermore, the perception of the DTM course was not measured and compared with the ITM course. The subjective students’ perception of the ITM course may be biased because the urge of the students to provide socially desirable answers that they thought the researchers wanted to hear. Another direction for future research may be to compare the ITM with other pedagogic methods like Flipped classroom.

## Supporting information

S1 Appendix DTM course contents. (DOC)

S2 Appendix DTM handouts. (DOCX)
