# Supplementary material for: Redesign and validation of a computer programming course using Inductive Teaching Method
Source: PLoS One. 2020 Jun 4;15(6):e0233716. doi: 10.1371/journal.pone.0233716 (PMC7272073; doi:10.1371/journal.pone.0233716)
Supplement: S1 Appendix — (DOC) [file pone.0233716.s001.doc]

# Course Plan Semester SP 2017

# Introduction to Computers and Programming

# Course Code: CSC 103

# Class: BSE-1A

# By: ---------

| Total Credit Hours: 4 | Lectures Credit Hours: 3 | Lab. Credit Hours: 1 |
| --- | --- | --- |
| Total Contact Hours: 6 | Lecture Contact Hours: 3 | Lab. Contact Hours:3 |

# **Office Hours** (Day, time and place): (9.00 am to 5.00 pm)

# **Pre-Requisite**: Nil

# **Course Objectives and Outcomes:**

| **First Part Objectives:** | The objective of this part is to familiarize the students with the computers, their history, type and applications in real life. Basic concepts of software and hardware will be taught to students in detail. By the end of first part the students will be totally familiar with the practical use of computer |
| --- | --- |
| **Second Part Objectives:** | After completion of the first part, the students will be able to understand basic concepts of computer programming. Which includes computer and computer Program, CPU level execution of program, Overview of programming Language, Different Levels of Programming language, Basic Structure and Elements of C Program, Input and Output, Variables, Data Types, Data Type Conversions, Format Specifiers, Escape Sequences, Arithmetic Operators, Arithmetic Assignment Operators, Precedence of Operators, Loops, Conditional statements, functions, Arrays etc. |
| **Ultimate Objectives:** | Computer programming is by its nature inherently mathematical learning a programming language is challenging and difficult and hard work for most students. However, upon completing this course the students should be able to outline and describe the basic concepts related to programming. Describe, analyze and use the various mechanisms in programming language, such as data types, scope, control structures subprograms, etc. |

# **Note:** The contents may be revised, if deemed necessary after the first Sessional test.

**Recommended Books:**

1. Turbo C Programming for the PC by Robert Lafore
2. A Book on C 4th Ed. By Al Kelley, Ira Pohl
3. C How to Program 4^th^ Edition, Dietel & Dietel

**Course Outline and Contents – Introduction to Computers and Programming**

| Week | **Lecture** | **Topic Covered** | **Date / Time Delivered** |
| --- | --- | --- | --- |
| **Week -1** | Lecture # 1 | Introduction to computer and computer Program, CPU level execution of Program, basics of Programming Language, Different Levels of Programming language, |  |
|  |  |  |  |
|  | Lecture # 2 | History of C Programming, Basic Structure and Elements of C Program, Input and Output, Variables, Data Types, Format Specifiers, Escape Sequences, Arithmetic Operators, Arithmetic Assignment Operators, Precedence |  |
|  |  |  |  |
| **Week -2** | Lecture # 3 | Relational Operators, Logical Operators, Precedence, Assignment Operator, postfix and prefix increment and decrement operators |  |
|  |  |  |  |
|  | Lecture # 4 | **Conditional Structures**: One-Way Selection Using the if-statement, Two-Way Selection Using the if…else statement, Relational and Logical operators use in Control Structures, with example programs |  |
|  |  |  |  |
| **Week - 3** | Lecture # 5 | Multiple Alternative Decision Using the Nested if statements, Multiple selection using the switch statement, Example Programs, with example programs  Flow charts, Standard library functions, Introduction to loops |  |
|  |  |  |  |
|  | Lecture # 6 | **Loops:** Introduction to Repetition Structures, Repetition essentials, counter controlled repletion, for repletion statement, examples using for statement, Nested for loops |  |
|  |  |  |  |
| **Week - 4** | Lecture # 7 | While statement, do while Repetition statement, break and continue statements, logical operators |  |
|  |  |  |  |
|  | Lecture # 8 | **Functions:** overview, function definitions, function prototypes |  |
|  |  |  |  |
| **Week - 5** | Lecture # 9 | Local and global variables |  |
|  |  |  |  |
|  | Lecture # 10 | **Arrays:** Overview, Declaring and referencing 1-D array, Using for Loops for Access of 1-D array elements, Array Examples |  |
|  |  |  |  |
| **Week - 6** | Lecture # 11 | Using Arrays with Functions, Referring to individual Elements of the Array |  |
|  |  |  |  |
|  | Lecture # 12 | Searching in 1-D Array |  |
|  |  |  |  |
| **Week - 7** | Lecture # 13 | **Strings:** Overview, Fundamentals of strings and characters, String Input/Output |  |
|  |  |  |  |
|  | Lecture # 14 | String manipulation functions of the string handling library, comparison functions of the string handling library, other functions |  |
|  |  |  |  |
| **Week - 8** | Lecture # 15 | **Pointers:** Overview, Pointer variable definition and initialization, Pointer Operators, Calling Functions by Reference |  |
|  |  |  |  |
|  | Lecture # 16 | Pointer expressions and pointer arithmetic, relationship between pointers and arrays, Pointers to functions |  |
|  |  |  |  |
| **Week - 9** | Lecture # 17 | 2-D Arrays |  |
|  |  |  |  |
|  | Lecture # 18 | **Structures:** Overview, structure definitions, initialization structures, accessing member of structures, |  |
|  |  |  |  |
| **Week - 10** | Lecture # 19 | using structures with functions |  |
|  |  |  |  |
|  | Lecture # 20 | Structures as parameters and return values of functions |  |
|  |  |  |  |
| **Week -11** | Lecture # 21 | **C Preprocessor:** #define directive symbolic Constants, #define directive macros etc. |  |
|  |  |  |  |
|  | Lecture # 22 | **Recursion:** concepts, with practical example program |  |
|  |  |  |  |
| **Week - 12** | Lecture # 23 | **Storage classes**: automatic storage class, static storage class, Register storage class and external storage class |  |
|  |  |  |  |
|  | Lecture # 24 | Enumerated data type, typedef, identifier and naming classes, type conversion |  |
|  |  |  |  |
| **Week - 13** | Lecture # 25 | **Disk Files:** Overview, What is the Need of Data File, Important Statements/Functions for handling I/O Data files |  |
|  |  |  |  |
|  | Lecture # 26 | Files and streams, creating a sequential access file, creating Random access files, Reading and writing in files |  |
|  |  |  |  |
| **Week -14** | Lecture # 27 | **C Data Structures**: Introduction |  |
|  |  |  |  |
|  | Lecture # 28 | **Graphics:** Basic of graphics |  |
|  |  |  |  |
| **Week-15** | Lecture # 29 | introduction of graphics functions |  |
|  |  |  |  |
|  | Lecture # 30 | **C++** : Basic Concepts (C++ vs C, C++ Classes, Data Abstraction etc) |  |
|  |  |  |  |
| **Week-16**  **Floating** | Lecture # 31 | **Slot for Sessional Test # 1 at the date and time as per semester schedule.** |  |
|  |  |  |  |
|  | Lecture # 32 | **Slot for Sessional Test # 2 at the date and time as per semester schedule.** |  |
|  |  |  |  |

**Rules and policy in this class**

1. 1. Although the rules and policy defined here may seem a little bit rush, they are not meant to cause any harm to you instead to protect you and to prepare you for better life and to become professionals.
2. 2. Student behavior/classroom decorum: “Free discussion, inquiry, and expression are encouraged this class.” However, classroom behavior that interferes with either the instructor’s ability to conduct the classroom or the ability of students to benefit from the instruction is not acceptable.
3. 3. Please turn off (or place on silence) your beepers and cellular phones before the lecture starts. In the event of a situation where student legitimately needs to carry a beeper/cellular telephone to class, prior notice and approval of the instructor is required.
4. 4. No use of electronic devices while in class unless required or approved by the instructor.
5. 5. Classroom behavior which is deemed inappropriate and cannot be resolved by the student and the faculty member may be referred to the Office of DCO for administrative or disciplinary review as per the code of Students Conduct.
6. 6. As part of the academic integrity outlined in the current General Catalogue: “Students are expected to maintain the highest standards of academic integrity. Behavior that violates these standards is not acceptable.
7. 7. Students are NOT allowed to share their assignments and to communicate during the tests or exam.
8. 8. No student is allowed in the class if not officially registered in this class.
9. 9. Late assignment will be penalized as follow after the assignment due date and time: 10% off the first day, 25% off the second day, 50% off the third day, and “Zero” after that. All the assignments will be due in class on the specified due date.
10. 10. No makeup test or exam will be given except in the case of emergency such as the student being sick and he/she is unable to come to class in which case an official Doctor’s excuse MUST be presented to the instructor. The student concerned is required to take the makeup test/exam no later than two lectures or class periods after he/she returns to class. Failure to comply will result in the grade of zero (0) for the test/exam.
11. 11. All students are encouraged to attend class and on time. I will be taking rolls randomly. A total of five (6) absences will result in the grade of “F” in this class for the student(s) concerned.
12. 12. It is totally forbidden to voice or/and video record this course lecture presentations without a written agreement signed and dated between the student and the instructor. Any violation to this rule will result to the invasion of the instructor privacy.
13. 13. Finally, I am totally open to any constructive critics or/and suggestions.
