# Supplementary material for: Redesign and validation of a computer programming course using Inductive Teaching Method
Source: PLoS One. 2020 Jun 4;15(6):e0233716. doi: 10.1371/journal.pone.0233716 (PMC7272073; doi:10.1371/journal.pone.0233716)
Supplement: S2 Appendix — (DOCX) [file pone.0233716.s002.docx]

**Time: 40 min Questions: 30** **Registration #:** ____________________

**Instructions:** Please read the following instruction carefully before attempting the paper.

1. There are MCQs in the test, each carrying equal marks. Each correct response gets 1 mark. Attempt all the questions.
2. For every question four or five options (A, B, C, D & E) are given, you have to select only one correct option.
3. Use ball point (black/blue) to shade the circle for correct option.
4. Erasing, cutting or overwriting is not allowed. Once an answer has been given on answer sheet, the candidate will not be allowed to change it. Shading two or more circles for a question will get Zero marks for that question.

**Part -1**

**Quantitative Reasoning (MCQs: 15)**

1. How many numbers consisting of two digits can be formed from 2, 3 5,7. Each integer is to be used only once.
2. 14
3. 12
4. 24
5. 8
6. 10
7. The next number in the pattern of 9; 10; 13; 18; …. is:
8. 21
9. 23
10. 25
11. 29
12. 19
13. The missing number in the following sequence is:


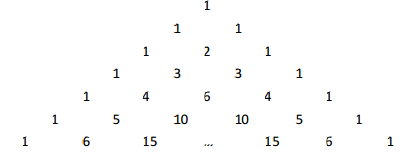


1. 5
2. 20
3. 21
4. 10
5. 425
6. What is 1/9 of 9?
7. A. 1/9
8. B. 0
9. C. 1
10. D. 2
11. The bar graph shows the results of a survey to what music people listen in the car.


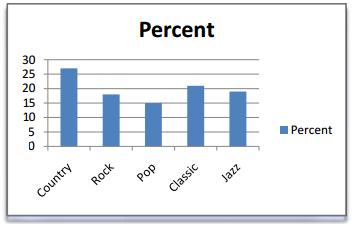


If you owned a store that specialized in car stereos, what type of music would you have playing?

1. Pop
2. Classic
3. Rock
4. Jazz
5. Country
6. Please find the figure continuing the series


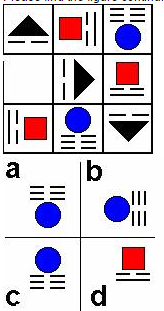


1. a
2. b
3. c
4. d
5. None of these
6. There are 24 birds on a tree. A hunter fired a gun and 20 birds fall down on ground. How many left on tree?
7. 4
8. 7
9. 20
10. 24
11. None
12. In the group off twenty girls Mary’s position is 7^th^ from the bottom what is the position of the Merry from top of the group?
13. 20^th^
14. 21^st^
15. 11^th^
16. 22^nds^
17. 19^th^
18. Which of the following fractions is equal to 5/6?

A. 20/30

B. 15/24

C. 25/30

D. 40/54

E. 2/7

1. The chart shows the number of symphony tickets sold by 11:00 on Thursday.


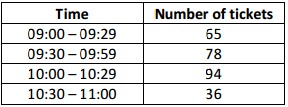


A. 143

B. 237

C. 273

D. 723

E. 73

1. The number of milliliters in 1 liter is

A. 10,000

B. 1,000

C. 0.1

D. 0.01

E. 0.001

1. A hockey team won 6 games and lost 8. What is the ratio of wins to number of games?

A. 6/8

B. 8/6

C. 3/7

D. 8/14

E. 6/7

1. Which of the following values is NOT equal to 34(58+9)?

A. 34 x 67

B. 58(34+9)

C. 34 x 58 + 34 x 9

D. 1,972 + 306

E. (9 + 58) 34

1. Which of the following Expression has the greatest value?
2. 3*3/3+3
3. 3*3+3+3
4. 3*3-3*3
5. 3/3+3*3
6. Which group of fractions is in descending order?
7. 2/3;3/4;5/8;5/6
8. 5/8;2/3;3/4;5/6
9. 2/3;3/4;5/6;5/8
10. 5/6;3/4;2/3;5/8
11. 2/3;1/2;3/4;4/5

**Part 2**

**Analytical Reasoning (MCQ’S 15)**

Questions 16-19

The supervisor of a commuter airline is schedul­ing pilots to fly the round-trip from City X to City Y. The trip takes only two hours, and the airline has one round-trip flight in the morning and one round-trip flight in the afternoon, each day, Monday through Friday. Pilots must be sched­uled in accordance with the following rules:

- Only W, X and Y can fly the morning flight.
- Only V, X and Z can fly the afternoon flight.
- No pilot may fly twice on the same day.
- No pilot may fly on two consecutive days.
- X must fly the Wednesday morning flight.
- Z must fly the Tuesday afternoon flight.

1. Which of the following must be true?
2. W flies the Monday morning flight
3. X flies the Monday afternoon flight
4. Y flies the Tuesday morning flight
5. W flies the Thursday morning flight
6. Z flies the Thursday afternoon flight
7. If X flies on Friday morning, which of the fol­lowing must be true?
8. X does not fly on Monday afternoon
9. V flies on Friday afternoon
10. W flies Thursday morning
11. Y flies Thursday morning
12. Neither W nor Y flies Thursday morning
13. If X flies only one morning flight during the week, which of the following must be true?
14. W flies exactly two days during the week
15. X flies exactly three days during the week
16. Y flies only one day during the week
17. Z flies Monday afternoon and Friday afternoon
18. X flies more times during the week than V
19. If W is not scheduled to fly at all during the week, all of the following must be true EXCEPT
20. X flies on Monday morning
21. V flies on Monday afternoon
22. Y flies on Thursday morning
23. Z flies on Friday afternoon
24. X flies on Friday morning

Questions 20-23

An obedience school is experimenting with a new training system. To test the system, three trainers (Luqman, Mehreen, and Omama) and three dogs (Lassie, Mugs, and Onyx) are assigned to three different rooms, one trainer, and one dog per room. The initial assignment is as follows:

Room 1: Luqman and Lassie

Room 2: Mehreen and Mugs

Room 3: Omama and Onyx

The participants have learned five different commands, each of which they will execute as soon as the command is given.

- Command W requires the trainer in Room 1 to move to Room 2, the trainer in Room 2 to move to Room 3, and the trainer in Room 3 to move to Room 1.
- Command X requires the dogs in Rooms 1 and 2 to change places.
- Command Y requires the dogs in Rooms 2 and 3 to change places.
- Command Z requires the dogs in Rooms 3 and 1 to change places.
- Command A requires each of the dogs to go to the room containing the trainer it was matched with in the initial assignment.

1. If the participants in the initial assignment are given exactly one command, Command W, which of the following will be true in the resulting arrangement?
2. Omama and Mugs will be in the same room
3. Mehreen will be in Room 3
4. Mehreen and Lassie will be in the same room
5. Luqman will be in Room 3
6. Luqman and Onyx will be in the same room
7. Which of the following commands or series of commands will yield a final arrangement in which Onyx is in Room 2?
8. One call of W
9. Two calls of X
10. Two calls of W followed by one call of A
11. Two calls of W followed by one call of Z
12. Two calls of X followed by one call of Z
13. Which of the following sequences of com­mands will yield a final arrangement in which Omama and Lassie are in Room 2?
14. X, Y, W
15. X, W, W
16. Z, W, A
17. X, Y, A, W
18. Z,W,W, X
19. Which of the following sequences of commands could result in a final arrangement in which Mehreen and Onyx are in Room 1, Omama and Mugs are in Room 2, and Luqman and Lassie are in Room 3?
20. Z, W, X
21. W, Y, Z
22. W, A, Y, W
23. W, Z, W, X
24. X, Z, W, W

Questions 24-25

All good athletes want to win, and all athletes who want to win eat a well-balanced diet; therefore, all athletes who do not eat a well-balanced diet are bad athletes.

1. Which of the following, if true, would refute the assumptions of the argument above?
2. Asma wants to win, but she is not a good athlete
3. Bashir, the accountant, eats a well-balanced diet, but he is not a good athlete
4. All the players on the Burhan baseball team eat a well-balanced diet
5. No athlete who does not eat a well-balanced diet wants to win
6. Chanda, the basketball star, does not eat a well-balanced diet, but she is a good athlete.
7. If the assumptions of the preceding argument are true, then which of the following statements must be true?
8. No bad athlete wants to win
9. No athlete who does not eat a well-balanced diet is a good athlete
10. Every athlete who eats a well-balanced diet is a good athlete
11. All athletes who want to win are good athletes
12. Some good athletes do not eat a well-balanced diet

Questions 26-29

Six students of foreign languages, Ameena, Bushra, Chand, Dilawer, Ehsan, and Farah, are seated together. They do not all speak the same language, but enough of them speak the same languages that they can translate for each other.

- Ameena and Dilawer speak only Urdu, Arabic and Hindi.
- Bushra speaks only Urdu, Arabic and Chinese.
- Chand speaks only Pushto and Hindi.
- Ehsan speaks only Hindi.
- Farah speaks only Chinese.

1. Which language is spoken by the most students?
2. Urdu
3. Arabic
4. Pushto
5. Hindi
6. Chinese
7. Which of the following students could talk to each other without a translator?
8. Ameena and Farah
9. Bushra and Chand
10. Bushra and Ehsan
11. Ehsan and Farah
12. Bushra and Farah
13. Who could act as a translator for a conversation between Bushra and Chand?
14. Ameena
15. Dilawer
16. Ehsan
17. Farah
18. I only
19. I & II
20. I, II & III
21. II, III & IV
22. I, II & IV
23. If Chand and Farah wish to talk to each other, what is the fewest number of translators they would need?
24. 0
25. 1
26. 2
27. 3
28. 4
29. Anne, Bongi and Carol are wearing dresses and shoes that are green, black or yellow. No two dresses or pairs of shoes are the same colour. Anne has yellow shoes. Bongi does not have a black dress or black shoes and only Carol has the same colour dress and shoes.

Bongi has

1. a green dress and yellow shoes
2. a black dress and green shoes
3. a green dress and green shoes
4. a green dress and black shoes
5. a yellow dress and green shoes

**Time: 50min**

**Read the instruction carefully and try to attempt all the examples.**

**Orientation**

Q1. Practice this program two times with different output.

**
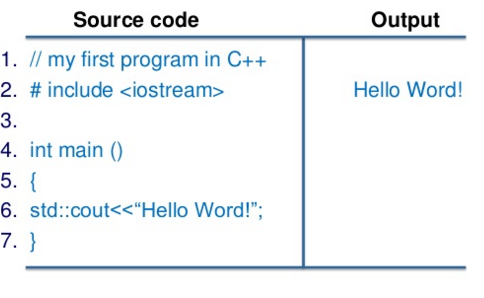
**

Q2. Write a simple program that prints your name. [Note: same method as mentioned in above example just write your name instead of hello word.]

Q3. Write a program that prints your registration number.

Q4. What will the following statements print? (Assume i=5 and j=7.)

a. cout << i << '\n';

b. cout << "i" << '\n';

c. cout << i / j << '\n';

d. cout << "i=" << i;

Q5. Practice this program into the tool.


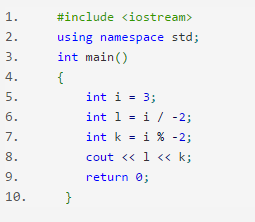


**Read the instruction carefully and try to attempt all the examples.**

**Variables:**

A variable provides us with named storage that our programs can manipulate.

**Try all the words mentioned below and identify correct variable names. If a given example give error, it is not a correct variable name. Try to figure out the rules for variable naming by comparing correct variable names with the incorrect variable names. Write down the rule you think could be operating in naming the variable in the blank space provided in the front of the word [Note: use keyword int before each name and use the semicolon at the end of each example like int abc; ]**

1. **name**
2. **Auto**
3. **False**
4. **max**
5. **MAX**
6. **main**
7. **45_min**
8. **float**
9. **if**
10. **_SUM**
11. **a**
12. **num^2**
13. **123**
14. **cin**
15. **Void**
16. **Inline**
17. **@**
18. **foo**
19. **NAME**
20. **098**
21. **INT**
22. **age**
23. **sum 1**
24. **x**
25. **657num**
26. **Cout**
27. **C_out**
28. **Z**
29. **1-2-3**
30. ***foo**

**2. Write the conclusion which you have drawn from above exercise.**

**1.**

**2.**

**3.**

**4.**

**5.**

**6.**

**7.**

**8.**

**9.**

**10.**

**Objective:** To get to know the correct way of naming variables. To identify that keywords could not be used to name variables and to find some basic errors related to naming variables

**Time: 50 min**

1. Try all the examples and identify the correct options.

Identify which variable initialization are correct and are not and write the reason you think could be for incorrect initialization. Note. Please take help from ‘error view’.

1. int abc = 10;
2. int sum_1 = 15;
3. int result = 90;
4. char a = ‘A’;
5. char name = 123;
6. char ch = ’123’;
7. char = ’var’;
8. int num1=”7abc”;
9. float num2=12.9;
10. char ch=’A’;
11. int=’M’;
12. float=”ali”;
13. char container=’A1’;
14. char container=’1A’;
15. char container=”A”;
16. float=12.5’;
17. int sum=50;
18. char ch=’B”;
19. float=”4.5”;
20. int x=250;
21. Try the following code and explain in the provided blank space what is happening. If nothing happens, try to enter some character from keyboard and press enter. Then write down your observations.
22. int i;

cin >> i;

1. int a;

cout<<”Enter the value of a\n”;

cin<<a;

1. char ch;

cin>>char;

1. float max:

cin>>max;

1. int a;

int b;

cin>>a>>b;

1. char name;

cin>>”name”;

1. int main;

cin>>main;

1. char c1;

char c2;

char c3;

cin>c1>>c2>>”c3”;

1. Try the following code and explain in the provided blank space what is output. Try to change the value of the variables and re-run the example again to observe and then write you observations in the provided space.
2. int a;

a=10;

cout<<”The value of a is”=<<a; ___________________________________________________

1. int a=10;

cout<<”The value of a is”=<<a;

What is the difference between statements of question 1 and 2. __________________________

The result of both statements equal or not? _________________________________________

1. char ch;

cin>>ch;

cout<<”The ch contains the character ”<<ch:

1. int a=5;

int b=10;

int sum;

sum=a+b;

cout<<”The sum of two variables is=”<<sum;

Practice the above program and rewrite the program in such a way that declare three variables and then get the value of variables from user and display the sum of variables.

1. Write a program that gives the bellow output.

Enter Two Numbers

3.4

2.3

Sum=5.7

1. Write a program to multiply and display the product of two numbers entered by user.

**Time: 50 min**

1. Execute the code below and observe. The code is to take input from the keyboard. Try code with different values i.e. integers (1,2,3…), decimal numbers i.e. floating or real numbers (1.0, 2.0, 3.5, …) or characters i.e. a, b, c. Write description of what each line of code is doing.
2. int i;

cin >> i;

1. int a;

cout<<”Enter the value of a\n”;

cin<<a;

1. char ch;

cin>>char;

1. float max:

cin>>max;

1. (i) int a; (ii) int a;

int b; int b;

cin>>a>>b; cin>>ab;

Which one is correct (i) or (ii) & why?

1. char name;

cin>>”name”;

1. char c1;

char c2;

char c3;

cin>c1>>c2>>”c3”;

1. Execute the code below and observe. The code is to view output on the computer screen. Write description of what each line of code is doing.
2. int a;

a=10;

cout<<”The value of a is”=<<a;

1. int a=10;

cout<<”The value of a is”=<<a;

What is the difference between statements of (i) and (ii).

Are both statements equal or not?

1. char ch;

cin>>”ch”;

cout<<”The ch contains\t ”<<ch:

1. int a=5;

int b=10;

int sum;

sum=a+b;

cout<<”The sum of two variables is=”<<sum;

Practice the above program and rewrite the program in such a way that declare three variables and then get the value of variables from the user and display the sum of the variables.

1. Write a program that gives following output.

Enter Two Numbers

3.4

2.3

Sum=5.7

1. Write a program to multiply and display the product of two numbers entered by user.

**Time: 50 min**

Try and execute the code below and observe. The code is used to take decisions. For example, if your age is greater than 18 years than you need to apply for an ID card. Write description of what each line of code is doing.

**Q1.** Write the following code as it is in your editor and execute it. Try the different values like: 18,27, 40, 69, 70, 71, 80, 90 etc. and observe the output.

int main()

{

int marks;

cout<<”Enter your marks”;

cin >> marks;

**if**(marks >= 70)

{

cout << "Wow,I am so happy!"<<endl;

}

cout << "Exit”;

return 0;

}

Output:

**Q2**. int x=9;

if(x>5);

cout<<”Number is 5”;

What you have observed? Output was “Number is 5” or nothing? Try the code again by removing ‘;’ semicolon from the front of if(x>5); Write your observation again.

**Q3**. Try following program by input your grandfather age. Dry different numbers up to 100.

[Suppose average age in Pakistan is above 65]

int main()

{

int age;

cout<<”My grandfather age is ”;

cin >> age;

**if**(age>65)

{

cout<<"Yes, My grandfather age is greater than the average age in Pakistan!";

}

if(age<=65){

cout << "No, My grandfather age is less than the average age in Pakistan!";

}

return 0;

}

Observations: ________________________________________________________________________________________________________________________________________________

**Q4.** What is the output of following condition?

int x=5; int y=10;

1. if(x>=y) cout << “X is greater than or equal to Y”;
2. if(x<=y) cout << “X is less than or equal to Y”;
3. if(x>y) cout << “X is greater than Y”;

Output: ---------------------------------------

Output: ---------------------------------------

Output: ---------------------------------------

**Time: 50 min**

Try and execute the code below and observe.

**Q1. Program to print integer entered by user only if that number is positive.**

#include <iostream>

using namespace std;

int main()

{

int number;

cout<< "Enter an integer: ";

cin>> number;

if ( number > 0) **// Checking whether an integer is positive or not.**

{

cout << "You entered a positive integer: "<<number<<endl;

}

{

cout<<"This statement is always executed because it's outside if statement.";

}

return 0;

}

1. **What is the output of the above program if you enter positive integer?**

**Output: ------------------------------------------------------------------------------------------------**

1. **What is the output if you enter negative integer?**

**Output: -------------------------------------------------------------------------------------------------**

1. **What is the output if you enter 0?**

**Output: ----------------------------------------------------------------------------------------------**

1. **What you think, in case of input number 0, the output is correct?**

**Output: ----------------------------------------------------------------------------------------------**

1. **If not correct, can you change the statement that need to be corrected?**

**Write correct statement below:**

**----------------------------------------------------------------------------------------------**

**Q2. Write and execute the following program and observe its output. What you think, its output is?**

#include <iostream>

using namespace std;

int main()

{

int number;

cout<< "Enter an integer: ";

cin>> number;

if ( number % 2 == 0) **// Checking whether an integer is EVEN or ODD.**

{

cout << "You entered an EVEN integer: "<<number<<endl;

}

{

cout<<"This is an ODD number.";

}

return 0;

}

1. **Try numbers 2,3,6,7,11,12 and write output below.**

**Output: ------------------------------------------------------------------------------------------------**

1. **What is the output if you enter 0?**

**Output: ----------------------------------------------------------------------------------------------**

1. **What you think, in case of ODD numbers, the output is correct?**

**Output: ----------------------------------------------------------------------------------------------**

1. **What you think, in case of EVEN numbers, the output is correct?**

**Output: ----------------------------------------------------------------------------------------------**

1. **If not correct, can you change the statement that need to be corrected?**

**Write correct statement below:**

**----------------------------------------------------------------------------------------------**

**Time: 50 min**

**Read the instruction carefully and try all the tasks in the VIP tool.**

**Q1. Enter the three numbers and find the maximum number from these three.**

// Finding the largest of 3 values

**Output**

#include <iostream>

int main()

{

int A, B, C, max;

cout << "Enter 3 values, and I will tell you which is largest.\n";

cin >> A >> B >> C; //really should have user prompts here

if (A > B)

max = A;

else

max = B;

if (C > max)

max = C;

cout << "The maximum is " << max << endl;

return 0;

}

**Q2.This Program is in pseudo code convert it in to C++ and see the result:**

**Hint: [grade >= 60]**

*If student’s grade is greater than or equal to 60*

*Print “Passed”*

*else*

*Print “Failed”*

**Q3.** Integers which are perfectly divisible by 2 are called even numbers. And those integers which are not perfectly divisible by 2 are not known as odd number. To check whether an integer is even or odd, the remainder is calculated when it is divided by 2 using modulus operator %. If remainder is zero, that integer is even if not that integer is odd. [Hint: condition (n%2 == 0) Correct program in lecture 7 by using an else].

Write a program for above scenario.

**Q4. Correct question 1, from lecture 7, using an else in the program**

**Q5. Alphabets a, e, i, o and u are vowels and all alphabets except these letters are consonants. This program below takes character input from user and checks whether that letter is vowel alphabet or not. [Here ‘c’ is a variable]**

#include <iostream>

using namespace std;

int main()

{

char c;

cout << "Enter an alphabet: ";

cin >> c;

if(c=='a'||c=='A'||c=='e'||c=='E'||c=='i'||c=='I'||c=='o'||c=='O'||c=='u'||c=='U')

{

cout << c << " is a vowel.";

}

Else

{

cout << c << " is not a vowel.";

}

return 0;

}

**Output_____________________________________________________________________**

**Time: 50 min**

**Q1. You have observed the vowels program in lecture 8. Here is another way to write this program. Write and execute this program. Try program with at least 10 different characters and with the combination of vowels, consonants and number etc. Write your observations.**

#include <iostream>

using namespace std;

int main ()

{

char ch; // local variable declaration:

cin >> ch;

switch(ch)

{

**case 'A' :**

**case 'a' :**

**case 'E' :**

**case 'e' :**

**case 'I' :**

**case 'i' :**

**case 'O' :**

**case 'o' :**

**case 'U' :**

**case 'u' :**

cout << “VOWELS”<<endl;

break;

**default**:

cout << "CONSONANTS or Other character or Number" << endl; }

} //end switch

return 0; }

**Observations ________________________________________________________________________________________________________________________________________________________________________________________________________________________________________________________________________________________________________________________**

**Q2. Try following program with all possible grades, Write the out of the program on after every execution.**

#include <iostream>

using namespace std;

int main ()

{

char grade; // local variable declaration:

cout<<”Please enter you grade to receive your message”<<endl;

cin >> grade;

switch(grade)

{

**case 'A' :**

cout << "Extraordinary. You won the battle. Keep up the good work" << endl;

break;

**case 'B' :**

cout << "V. Good. You are about to win the battle. Keep trying" << endl;

break;

**case 'C' :**

cout << "Satisfactory. You need to work hard to win the battle" << endl;

break;

**case 'D' :**

cout << "Fair. You were about to lose battle. Don’t stand here. Do something." << endl;

break;

**case 'F' :**

cout << "Poor. You lost the battle. But don’t give up. Try again and come with greater zeal." << endl;

break;

default :

cout << "This grade did not exist yet. Please enter a correct grade from A,B,C,D, F" << endl;

} //end switch

cout << "Your grade is " << grade << endl;

return 0;

} //end main

**Output______________________________________________________________________________________________________________________________________________________**

**______________________________________________________________________________**

**Q3. C++ program to build simple calculator using switch Statement. Execute the program by using different operators and write output. Extend the calculator for some other mathematical operations as well. I.e. for remainders use ‘%’;**

#include <iostream>

using namespace std;

int main() {

char operator;

float num1,num2;

cout<<"Select an operator either ‘+’ or ‘-‘ or ‘*’ or ‘/’ \n";

cin>>operator;

cout<<"Enter two operands: ";

cin>>num1>>num2;

switch(operator) {

**case '+':**

cout<<num1<<" + "<<num2<<" = "<<num1+num2;

break;

**case '-':**

cout<<num1<<" - "<<num2<<" = "<<num1-num2;

break;

**case '*':**

cout<<num1<<" * "<<num2<<" = "<<num1*num2;

break;

**case '/':**

cout<<num1<<" / "<<num2<<" = "<<num1/num2;

break;

**default**:

printf("Error! operator is not correct");

} // end switch

return 0;

} //end main

**Output _________________________________________________________________**

**Time: 50 min**

Q1. Try these programs and write the output of each. Identify the difference between each example.

1. **#include<iostream> // header file**

**using namespace std;**

**int main()**

**{**

**int a = 10;
 a++; //increment by 1
 cout << a;**

**return 0;**

**}**

1. **#include<iostream> // header file**

**using namespace std;**

**int main()**

**{**

**int a = 10;
 a=a+1;
 cout << a;**

**return 0;**

**}**

**What is the output of the above programs?**

**Are both programs giving the same result?**

**--------------------------------------------------------**

**--------------------------------------------------------**

**--------------------------------------------------------**

1. **#include<iostream> // header file**

**using namespace std;**

**int main()**

**{**

**int a=25;**

**int b;**

**b=a++; // assignment first?**

**cout<<b;**

**return 0;**

**}**

1. **#include<iostream> // header file**

**using namespace std;**

**int main()**

**{**

**int a=25;**

**int b;**

**b=a+1;**

**cout<<b;**

**return 0;**

**}**

**What is the output of above program?**

**-------------------------------------------------------------**

**Which variable value will be displayed in the above program?**

**-------------------------------------------------------------**

**In case of program c, the value assignment occurred first or increment was first.**

**-------------------------------------------------------------**

**What if we change the statement line 33 as**

**b = ++a;**

**What will be output now?**

**Which operator will take priority now? ‘=’ or ‘++’**

**______________________________________**

**Decrement Operators**

Q2. Try these programs and write the output of each.

And identify the difference between each example.

1. **#include<iostream> // header file**

**using namespace std;**

**int main()**

**{**

**int a = 10;
 a--;
 cout << a;**

**return 0;**

**}**

1. **#include<iostream> // header file**

**using namespace std;**

**int main()**

**{**

**int a = 10;
 a=a-1;
 cout << a;**

**return 0;**

**}**

**What is the output of the above programs?**

**Is both programs give the same result?**

**--------------------------------------------------------**

**--------------------------------------------------------**

**--------------------------------------------------------**

1. **#include<iostream> // header file**

**using namespace std;**

**int main()**

**{**

**int a=25;**

**int b;**

**b=a--; //assignment first?**

**cout<<b;**

**return 0;**

**}**

1. **#include<iostream> // header file**

**using namespace std;**

**int main()**

**{**

**int a=25;**

**int b;**

**b=a-1;**

**cout<<b;**

**return 0;**

**}**

**What is the output of above program?**

**-------------------------------------------------------------**

**Which variable value will be displayed in the above program?**

**-------------------------------------------------------------**

**In case of program c, the value assignment occurred first or decrement was first.**

**What if we change the statement line 98 as**

**b = --a;**

**What will be output now?**

**Which operator will take priority now? ‘=’ or ‘--’**

**______________________________________**

**Time: 50 min**

1. **Write a program that will print first ten integer in descending order.**

**[Hint: use decrement operator --]**

Program:

Output: _____________________________

**Q2.** **Write a program using for loop that will calculate the sum of the integers between 1 and 20.**

#include <iostream>

using namespace std;

int main(){

int sum=0;

int count ;

for (count=1; count<=20; count++)

sum = sum + count;

cout << "The sum of the integers from 1 through 20 is = ";

cout << sum << endl;

return 0;

**}**

**Output:**_______________________________

**Registration #:** ___________________

1. **Input first five numbers and find the average of the numbers using for loop.**

**[Hint: formula to find the average is:**

**average = sum of number / total numbers ]**

Program:

**Output**:________________________________

_______________________________________

**Name:**  **Registration #:**

**Read the instruction carefully and according to instruction attempt all the tasks.**

- 1. **What is the output of the below program?3. This program displays the prime numbers from 2 to 50 using nested loop.**

#include <iostream> using namespace std; int main()

{

int N = 5; int M = 1;

for (int i = 0; i < N; i++)

{

for (int j = 1; j <= M; j++)

{

cout << j;

}

cout << endl; M++;

}

return 0; }

#include <iostream> using namespace std; int main ()

{

int i,j;

int count = 0; for(i=2; i<50; i++)

{

for(j=1; j <= 50; j++)

{

if(i%j==0)
 count++;

Output:

**2. Write a program that prints the below output:**

12345
1234
123
12
1}

// if factor found, not prime if(count == 2)

cout << i << " is prime\n"; count = 0;

}

return 0;}

Output:

**Time: 50 min**

3. A) Convert it into C++ program and execute it.

B) Write the output of the program.


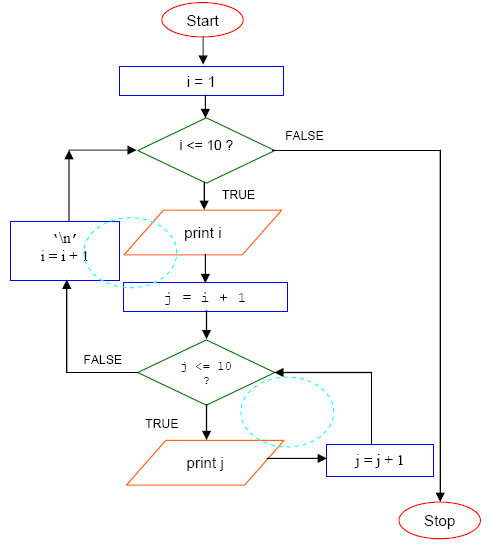


**Time: 50 min**

Q1. Write a program using while loop that calculate the sum of all the digits from 1 to given number?


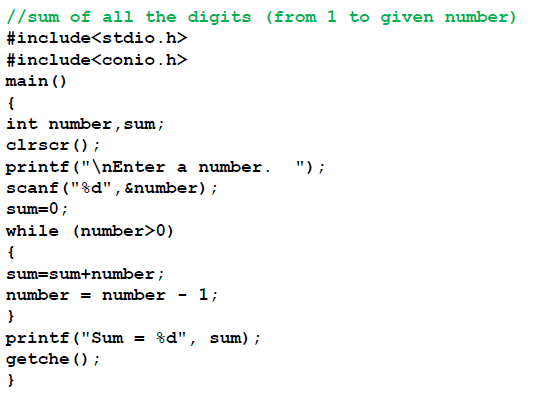


Practice this program and write the output.

Q2.A series of number in which each number is the sum of preceding two numbers is known as Fibonacci series.

1, 1, 2, 3, 5, 8, 13, 21, 34...

In this example, user is asked to enter a positive integer (Suppose *n*) and Fibonacci series is displayed up to *n*th term.

Fibonacci sequence up to *n*th term

1. **#include <iostream>**
2. **using namespace std;**
3. **int main() {**
4. **int n, firstTerm = 1, secondTerm = 1, nextTerm;**
5. **cout << "Enter number of terms: ";**
6. **cin >> n;**
7. **cout << "Fibonacci Series: " << firstTerm << " + " << secondTerm << " + ";**
8. **for (int i = 1; i <= n-2; ++i) {**
9. **nextTerm = firstTerm + secondTerm;**
10. **cout << nextTerm << " + ";**
11. **firstTerm = secondTerm;**
12. **secondTerm = nextTerm;**
13. **}**
14. **return 0;**
15. **}**

Output

Enter number of terms: 11

Fibonacci Series: 1 + 1 + 2 + 3 + 5 + 8 + 13 + 21 + 34 + 55 + 89 +

Q5. Find the LCM by using do while loop.

1. #include <iostream>

**Output:**

**Enter two numbers: 12**

**18**

**LCM = 36**

1. using namespace std;
2. int main() {
3. int n1, n2, max;
4. cout << "Enter two numbers: ";
5. cin >> n1 >> n2;
6. max = (n1 > n2) ? n1 : n2; // maximum value between n1 and n2 is stored in max
7. do {
8. if (max%n1 == 0 && max%n2 == 0) {
9. cout << "LCM = " << max;
10. break;
11. }
12. else
13. ++max;
14. }
15. while (true);
16. return 0;

}

Q6. Simple program to run do while loop.

Output

value of i : 0

value of i : 1

value of i : 2

value of i : 3

value of i : 4

value of i : 5

value of i : 6

value of i : 7

value of i : 8

1. #include <iostream>
2. using namespace std;
3. int main ()
4. {
5. s// Local variable declaration:
6. int i = 0;
7. // do loop execution
8. do
9. {
10. cout << "value of i : " << i << endl;
11. i++;
12. }
13. while( i < 10 );
14. return 0;
15. }

**Time: 50 min**

**Q1. Enter the value of two variables on the output screen and display the product of the input numbers.**

**#include<iostream> // header file**

**using namespace std;**

**int main()**

**{**

**int number1; //variable declaration**

**int number2; //variable declaration**

**int product;     //variable declaration**

**cout<<"enter the value of number1 :";  /* Display the message to tell the**

**user to do appropriate action */**

**cin>>number1;  //Get the value of number1**

**cout<<"enter the value of number2 :"; /* Display the message to tell the**

**user to do appropriate action */**

**cin>>number2; //Get the value of number2**

**product=number1*number2; //calculate the product**

**cout<<"the product of number1 & number2 is "<<product;  //display the Product**

**return 0;**

**}**

**Q2. Enter the value of two variables on the output screen and display the sum, subtraction and remainder.**

**Time: 50 min**

**i) Draw a flow diagram of below program.**

// custom countdown using while

#include <iostream>

using namespace std;

int main ()

{

int n =1;

while (n<=10) {

cout << n << ", ";

n++;

}

cout << "print end of program!\n";

}

return 0;

}

Flow diagram

1. **i)** **Convert the below pseudocode in to C++ program.**

Initialize a number

While number is greater than 0

Print the number

Decrease the number by 1

End of while loop

Print “I am good programmer”

Write the program here

**ii)** Write the output of the program.

_____________________________________

1. **Convert this flow chart into C++ program and write the output.**


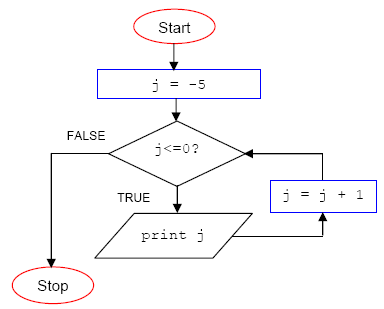


| **Week** | **Lecture** | **Topic covered** | **Objective** |
| --- | --- | --- | --- |
| **Week-1** | **Lecture 1**  G. Orientation  &  Pre-test | - Class rules, introduction to computer and programing, History of languages, types of languages and IDEs. - 30 question basic mathematics,   30 question analytical reasoning. | - Students will know the class rules, importance of attendance and programing criteria. - To measure the mathematical skills of the students. |
|  | **Lecture 2**  Introduction to computers | Introduction to the basic units of computer, overview of evolution and advancements in the basic units of computers, introduction to CPU, how CPU function, basic instruction execution, functionalities of different types of memory. | Students will be able to know the basic components of computers and functionality of each component. |
| **Week-2** | **Lecture 3**  Introduction to computers | Introduction to number system, introduction to storage media, primary storage devices, secondary storage devices, how data is stored. | How number system works, how primary and secondary storage devices store data. |
|  | **Lecture 4**  File system | Evaluation of file system, how file systems works, optical storage devices and solid-state storage devices. | Students will be able to know what are files and how they work. |
|  | **Lecture 5 (lab)**  S. Orientation | Basic structure and elements of C++ program, input, output, escape sequences, arithmetic operators, operator precedence. | Students will be able to understand the basic concept of the computer programing. |
| **Week-3** | **Lecture 6**  Program and software | Introduction and differentiation between program and software, intro to different types of software, introduction to operating system. | Differentiate program and software and know about the operating systems. |
|  | **Lecture 7**  Computer and program | Introduction to computer and program, CPU level execution of program, basics of programing languages, basic structures and execution of C++ program. | Students will be able to know CPU level execution of program, basics of programing. |
|  | **Lecture 8 (lab)**  Variables | Variables types, Declaration of variable, Multiple declaration of variables, reserved keywords.  data types, Assignments of variable. | How variables works in computer memory and programing.  How to declare variables. Students will identified the rules to declare variables.  How to initialize it, and identified the different data types.  Students will be able to know how to handle multiple cases, logic of the operators used in conditional statements. |
| **Week-4** | **Lecture 9** | One-way selection using if-statement. Conditional operators, Logical and rational operators. | Students will be able to know the conditional structures, simple one-way selection and operators. |
|  | **Lecture 10** | Nested if statement,  Two-way selection using if-else statement | Students will be able to know the conditional structures, simple two-way selection and operators. |
|  | **Lecture 11 (lab)**  Conditional-structure | One-way selection using if-statement. Conditional operators, Logical and rational operators. | Students will be able to know the conditional structures, simple one-way selection and operators. |
| **sWeek-5** | **Lecture 12**  **Post-test1** | Included lecture 1 to 11 | To assess the students conceptual knowledge. |
|  | **Lecture 13** | Multiple-selection using the switch statement.  Different examples programs. | How to select multiple statements by using switch statement. |
|  | **Lecture 14 (lab)**  Conditional-Structure | Two-way selection using if-else statement.  Multiple-selection using the switch statement. | Students will be able to know the conditional structures, simple two-way selection and operators. |
| **week-6** | **Lecture 15** | Revision |  |
|  | **Lecture 16** | Revision |  |
|  | **Lecture 17(lab)**  **Practical-task1** | Included Lecture 8, Lecture 11, Lecture 14 | To assess the student problem solving skills. |
| **Week-7** | **Lecture 18** | Introduction to repetition structures, repetition essentials,  Counter controlled repetition. | How counter controls works. |
|  | **Lecture 19** | for loop, structure of for loop, examples of for loop. Nested for loop. | How for loop structure works. |
|  | **Lecture 20 (lab)**  Loops (for) | Introduction to repetition structures, repetition essentials, for statement, examples. | How for loop structure works. |
| **Week-8** | **Lecture 21** | while loop, structure of while loop, examples of while loop. Use of logical operators in while loop. | How while loop structures works. |
|  | **Lecture 22** | Do-while loop with example.  Break and continue statement. | How do-while loop structures works. |
|  | **Lecture 23**  Loops  While & do-while | do-while statement, break and continue statement. | How while and do-while loop structures works. |
| **Week-9** | **Lecture 24**  **Post-test2** | Lecture 15 to 23 | To assess the students’ conceptual knowledge. |
|  | **Lecture 25** | **Functions:** introduction and overview, function definitions, function prototypes. | Students will able to define the functions |
|  | **Lecture 26**  **Practical-task 2** | Included lecture 20, lecture 23 | To assess the student problem solving skills |
| **Week-10** | **Lecture 27** | **Arrays:** overview, declaring and referencing 1-D array, using for loops for accessing 1-D array elements, programming examples of arrays. | Students will be able to Search and sort the array elements insertion & deletion in 1-D Array. |
|  | **Lecture 28** | Using arrays with functions, referring to individual elements of the array with programming examples. | Students will be able to create arrays of different types. |
| **Week-11** | **Lecture 29** | 2-D arrays in detail with examples and applications. | Knowledge about different arrays and there applications. |
|  | **Lecture 30** | **Strings:** overview, fundamentals of strings and characters, string input/output. | Students know that how strings and characters are define. |
| **Week-12** | **Lecture 28** | String manipulation and comparison functions of the string handling library and other functions. | Students know that how to manipulate and compares the string functions. |
|  | **Lecture 31** | **Pointers:** overview of pointers, pointer variable definition and initialization, pointer operators, calling functions by reference | How pointers works in the memory and how they declare and initialized. |
| **Week-13** | **Lecture 32** | Pointer expressions and pointer arithmetic, relationship between pointers and arrays, using pointers with functions | Students have knowledge about relationships between pointers and arrays. |
|  | **Lecture 33** | **Structures:** overview and introduction, structure definitions, initialization of structures, accessing members of structures. | Students will be able to define  structure, initialization of structures, accessing members of structures. |
| **Week-14** | **Lecture 34** | Using structures with functions and arrays. | Uses of structures. |
|  | **Lecture 35** | Structures as parameters and return values of functions, some example applications. | Students will be able to present  Example applications of structures. |
| **Week-15** | **Lecture 36** | **Recursion:** concept with practical examples. | Students will be able to define recursive functions. |
|  | **Lecture 37** | **C Preprocessor:** #define directive symbolic constants, #define directive macros etc. | How Preprocessor works. |
| **Week-16** | **Lecture 38** | Semester Project Discussion – Student Presentations. | To prepare students for the final projects. |
|  | **Lecture 39** | Sample Project and course revision. |  |

**Post-test one for assessing the students’ knowledge about programing course after four weeks of study.**

Name: Registration #:

This test contains 30 items. Read carefully and select only one suitable option.

1. Programs are converted into the machine language with the help of?

A An editor

B. Compiler

C. An operating system

D. None of the above

1. C++ can be used on?

A. Only MS-DOS

B. Only Linux

C. Only window

D. All of the above

1. C++ language has been developed by?

A. Ken Thompson

B. Dennis Ritchie

C. Peter Norton

D. None of above

1. C++ variable cannot start with?

A. An alphabet

B. A number

C. A special symbol other than underscore

D. Both (2) and (3)

1. A character variable can at a time store?

A. 1 character

B. 8 characters

C. 254 characters

D. None of the above

1. Which of the following is not a character constant?

A. 'Thank You'

B. 'quest videos- IT learning at its best'

C. '23.56e-03'

D. All of the above

1. Which of the following statements is wrong?

A. INT=123;

B. Val='A' * 'B';

C. Is=20 * 'T'

D. Count+5=res;

1. What punctuation ends most lines of C++ code?

A. . (dot)

B. ; (semi-colon)

C. : (colon)

D. ' (single quote)

1. Which of the following is not a correct variable type?

A. Float

B. Real

C. Int

D. Double

1. Which of the following is a correct comment?

A. */ Comments */

B. ** Comment **

C. /* Comment */

D. { Comment }

1. Which of the following cannot be used as identifiers?

A. Letters

B. Digits

C. Underscores

D. Spaces

1. Which of the following is not a valid escape code?

A.\t

B.\v

C.\f

D)\w

1. Which of the following is allowed in a C Arithmetic Instruction?

A) [ ]

B) { }

C) ( )

D) None of the above

1. To accept 100 different values into the array we require

A) Loop

B) If condition

C) Function

D) Structure

1. If a is an integer variable, a=7/3; will return a value

A) 2.5

B) 3

C) 0

D) 2

1. What is the result of following statement

x=y=z=0;

1. x=0, y=Null, z=Null
2. x=0, y=0, z=0
3. x=0, y=1, z=2
4. the statement is incorrect
5. Which one of the following is not a reserved keyword for C?
6. auto
7. case
8. main
9. default
10. A C variable cannot start with
11. A number
12. A special symbol other than underscore
13. Both of the above
14. Both of the above
15. What is the correct value to return to the operating system upon the successful completion of a program?

## 1

## -1

## 0

## Program do no return a value

## Which is the only function all C programs must contain?

1. start()
2. system()
3. main()
4. printf()

## Which of the following is not a correct variable type?

## float

## real

1. int
2. double

## Which of following is not a valid name for a C++ variable?

## Examveda

## Exam veda

## Both A and B

## None of these

## What is the difference between a declaration and a definition of a variable?

1. Both can occur multiple times, but a declaration must occur first.
2. A definition occurs once, but a declaration may occur many times.
3. A declaration occurs once, but a definition may occur many times
4. Both can occur multiple times, but a definition must occur first
5. Which of the following statements are correct about an if-else statements in a C-program?

| 1: | Every if-else statement can be replaced by an equivalent statements using   ?:operators |
| --- | --- |
| 2: | Nested if-else statements are allowed. |
| 3: | Multiple statements in an if block are allowed. |
| 4: | Multiple statements in an else block are allowed. |

1. 1 and 2
2. 2 and 3
3. 1, 2, 4
4. 2, 3, 4
5. Which of the following correctly shows the hierarchy of arithmetic operations in C?
6. / + * -
7. *- / +
8. + - / *
9. / * + -
10. In which order do the following gets evaluated

| 1. | Relational |
| --- | --- |
| 2. | Arithmetic |
| 3. | Logical |
| 4. | Assignment |

1. 2134
2. 1234
3. 4321
4. None of above
5. Which of the following is the correct order of evaluation for the below expression?
   z = x + y * z / 4 % 2 – 1
6. / % + - =
7. = * / % + -
8. / * % - + =
9. % / - + =

## Find the output of the following program. void main() { int i=01289; printf("%d", i); }

## 0289

## 1289

## 713

## Syntax error

1. Which of the following is known as insertion operator?
2. ^
3. v
4. <<
5. >>
6. Which of the following is output statement in C++?
7. print
8. write
9. cout
10. cin

**Post-test for assessing the students’ knowledge about programing course.**

Name: Registration #:

Q1.This test contains 30 items. Read carefully and select only one suitable option.

1. Which looping process checks the test condition at the end of the loop?
2. for
3. while
4. do-while
5. no looping process checks the test condition at the end
6. When following piece of code is executed, what happens?

b = 3;

a = b++;

1. a contains 3 and b contains 4
2. a contains 4 and b contains 4
3. a contains 4 and b contains 3
4. a contains 3 and b contains 3
5. What is the final value of x when the code int x; for(x=0; x<10; x++) {} is run?

A. 10

B. 9

C. 0

D. 1

1. When does the code block following while(x<100) execute?

A. When x is less than one hundred

B. When x is greater than one hundred

C. When x is equal to one hundred

D. While it wishes

1. Which is not a loop structure?

A. for

B. do while

C. while

D. repeat until

1. How many times is a do while loop guaranteed to loop?

A. 0

B. Infinitely

C. 1

D. Variable

1. Each pass through a loop is called a/an
2. enumeration
3. iteration
4. culmination
5. pass through
6. A continue statement causes execution to skip to
7. the return 0; statement
8. the first statement after the loop
9. the statement following the continue statement
10. the next iteration of the loop
11. In a group of nested loops, which loop is executed the most number of times?
12. the outermost loop
13. the innermost loop
14. all loops are executed the same number of times
15. cannot be determined without knowing the size of the loops
16. Which looping process is best used when the number of iterations is known?
17. for
18. while
19. do-while
20. all looping processes require that the iterations be known
21. What's wrong? for (int k = 2, k <=12, k++)
22. the increment should always be ++k
23. the variable must always be the letter i when using a for loop
24. there should be a semicolon at the end of the statement
25. the commas should be semicolons
26. If there is more than one statement in the block of a for loop, which of the following must be placed at the beginning and the ending of the loop block?
27. parentheses ( )
28. braces { }
29. brackets [ ]
30. arrows < >
31. The difference between while structure and do structure for looping is
32. In while statement the condition is tested at the end of first iteration
33. In do structure the condition is tested at the beginning of first iteration
34. The do structure decides whether to start the loop code or not whereas while statement decides whether to repeat the code or not
35. In while structure condition is tested before executing statements inside loop whereas in do structure condition is tested before repeating the statements inside loop
36. Which of the following is not a looping statement in C?
37. while
38. until
39. do
40. for
41. The continue statement
42. resumes the program if it is hanged
43. resumes the program if it was break was applied
44. skips the rest of the loop in current iteration
45. all of above
46. Observe the following block of code and determine what happens when x=2?

switch (x){

case 1:

case 2:

case 3:

 cout<< "x is 3, so jumping to third branch";

goto third Branch;

default:

 cout<<"x is not within the range, so need to say Thank You!";

                }

1. Program jumps to the end of switch statement since there is nothing to do for x=2
2. The code inside default will run since there is no task for x=2, so, default task is run
3. Will display x is 3, so jumping to third branch and jumps to third Branch.
4. None of above
5. Which of the following is false for switch statement in C++?
6. It uses labels instead of blocks
7. we need to put break statement at the end of the group of statement of a condition
8. we can put range for case such as case 1..3
9. None of above
10. Looping in a program means
11. Jumping to the specified branch of program
12. Repeat the specified lines of code
13. Both of above
14. None of above
15. Find out the error in following block of code.

if (x = 100)

cout << “x is 100”;

1. 100 should be enclosed in quotations
2. There is no semicolon at the end of first line
3. Equals to operator mistake
4. Variable x should not be inside quotation
5. Consider
6. int a = 6;
7. int b = 12;
8. while(a<b)
9. {
10. cout<<"In the loop";
11. a+=2;
12. b-=2;
13. }

How many times is the phrase "In the loop" printed?

A. 1

B. 2

C. 3

D. 4

E. 5

1. Consider the following piece of code:
2. int i;
3. for(i=0; i<10; i++){
4. /* some code that doesn't modify i */
5. }
6. cout<<"i="++i; /* line A */
7. What is printed by the statement on line A?
8. i=0
9. i=9
10. i=10
11. i=11
12. This piece of code doesn't compile since the scope of i is limited

to the for loop.

1. What is the output of this program?
2. #include <iostream>
3. using namespace std;
4. int main()
5. {
6. int i;
7. for (i = 0; i < 10; i++);
8. {
9. cout << i;
10. }
11. return 0;
12. }
13. 0123456789
14. 10
15. 012345678910
16. compile time error
17. How many types of loops are there?
18. 4
19. 2
20. 3
21. 1
22. How many sequence of statements are present in c++?
23. 4
24. 3
25. 5
26. 6
27. The if-else statement can be replaced by which operator?
28. Bitwise operator
29. Conditional operator
30. Multiplicative operator
31. none of the mentioned
32. Which of the following statements about the while loop is not true?
33. a.The while loop is a posttest loop.
34. b.Testing condition is made before each iteration.
35. c.The while loop statement must terminate with a semi-colon.
36. None of them
37. The first expression in a for loop is
38. Step value of loop
39. Value of the counter variable
40. Any of above
41. None of above
42. What is the final value of x if initially x has the value 1?

if (x >= 0)

x += 5;

else if (x >=5)

x += 2;

1. 8
2. 6
3. 1
4. 4

**Section B**

Q.2Which of the following while statements is equivalent to

1. do{
2. y=x+7;
3. x++;
4. }while(x<9);

while(x<9)

{

y=x+7;

x++;

}

y=x+7;

x++;

while(x<=9)

{

y=x+7;

x++;

}

y=x+7;

x++;

while(x<9)

{

y=x+7;

x++;

}

Q.3 Change the following C++ code from a while loop to a for loop:

int x;

cin>>x;

while(x!=10)

{

cout<<x<<"\t";

cin>>x;

}

Q4. Which of the followings is not a nested loop?

1. for(i=0;i<10;i++)

for(j=1;j<i+2;j++)

2. for(i=0;i<10;i++)

cout<<"i="<<i;

for(j=1;j<i+2;j++) cout<<"j="<<j;

3. for(i=0;i<10;i++)

while(j%2!=0){ cout<<j<<"\t";j++;}

Q5. If originally x=1,y=0, and z=1, what is the value of x, y, and z after executing the following code?

if(x>y && x>z) {y=x;z=x+1;}

else if(x+y>=z) {x++;z=x+1;}

else y=z+x;

Q6. Write a program in c++ that read a number from user print its reverse number. Using while loop.

**Section C**

Q7. Write a nested if-else statement that will assign a character grade to a percentage mark as follows - 70 or over A, 60-69 B, 50-59 C, 40-49 D, 30-39 E, less than 30 F.

Q8.Write a program that prints a right angle triangle using for loop

Q9.Write a program that find Fibonacci series with simple logic

Q10.Write a program to find greatest number between 3 number using if-else-if statement.

Q.11 Write a program to print number from 1 to 10.

Name: Registration #:

This paper contains three sections A, B, and C

**Section A**

This section contains 21 items. Read carefully and select only one suitable option. Each question carries 1 mark. Erasing, cutting or overwriting is not allowed. Selecting two or more options for a question will get zero marks for that question.

Q1. This test contains 21 items. Read carefully and select only one suitable option.

1. In which loop the test condition is checked at the end?
2. for
3. while
4. do-while
5. no loop
6. When following piece of code is executed, what happens?

**b = 3;**

**a = b++;**

1. a contains 3 and b contains 4
2. a contains 4 and b contains 4
3. a contains 4 and b contains 3
4. a contains 3 and b contains 3
5. What is the final value of x when the code

**int x;**

**for(x=0; x<10; x++) {} is executed?**

A. 10

B. 9

C. 0

D. 1

1. When does the code block following while(x<100) execute?

A. When x is less than one hundred

B. When x is greater than one hundred

C. When x is equal to one hundred

D. While it wishes

1. Which is not a loop structure?

A. for

B. do while

C. while

D. repeat until

1. How many times is a do while loop guaranteed to loop?

A. 0

B. Infinitely

C. 1

D. Variable

1. Each pass through a loop is called a/an
2. enumeration
3. iteration
4. culmination
5. pass through
6. In a group of nested loops, which loop is executed most number of times?
7. the outermost loop
8. the innermost loop
9. all loops are executed the same number of times
10. cannot be determined without knowing the size of the loops
11. What's wrong? **for (int k = 2, k <=12, k++)**
12. the increment should always be ++k
13. the variable must always be the letter i when using a for loop
14. there should be a semicolon at the end of the statement
15. the commas should be semicolons
16. If there is more than one statement in the block of a for loop, which of the following must be placed at the beginning and the ending of the loop block?
17. parentheses ( )
18. braces { }
19. brackets [ ]
20. arrows < >
21. The difference between while structure and do structure for looping is
22. In while statement the condition is tested at the end of first iteration
23. In do structure the condition is tested at the beginning of first iteration
24. The do structure decides whether to start the loop code or not whereas while statement decides whether to repeat the code or not
25. In while structure condition is tested before executing statements inside loop whereas in
26. do structure condition is tested before repeating the statements inside loop
27. Observe the following block of code and determine what happens when x=2?

**switch (x){**

**case 1:**

**case 2:**

**case 3:**

**cout<< "x is 3, so jumping to third branch";**

**default:**

**cout<<"x is not within the range, so need to say Thank You!";**

**}**

1. Program jumps to the end of switch statement since there is nothing to do for x=2
2. The code inside default will run since there is no task for x=2, so, default task is run
3. Both cout statements will execute.
4. None of above
5. In switch statement, the test condition can contain the logical and relational operators
   1. True
   2. False
   3. Both A & B
   4. None of the A & B
6. Loop is used for
7. Branching
8. Repetition
9. Both of above
10. None of above
11. Find out the error in following block of code.

**if (x = 100) {**

**cout << “x is 100”;}**

1. 100 should be enclosed in quotations
2. There is no semicolon at the end of first line
3. Equals to operator mistake
4. Variable x should not be inside quotation
5. Consider

**int a = 6;**

**int b = 12;**

**while(a<b)**

**{**

**cout<<"In the loop";**

**a+=2;**

**b-=2;**

**}**

How many times is the phrase "In the loop" printed?

A. 1

B. 2

C. 3

D. 4

E. 5

1. Consider the following piece of code:

**int i;**

**for(i=0; i<10; i++){**

**/* some code that doesn't modify i */**

**}**

**cout<<"i="<<++i; /* line A */**

What is printed by the statement on line A?

1. i=0
2. i=9
3. i=10
4. i=11
5. This piece of code doesn't compile since the scope of i is limited to the for loop.
6. What is the output of this program?

**int main()**

**{**

**int i;**

**for (i = 0; i < 10; i++);**

**{**

**cout << i;**

**}**

**return 0;**

**}**

1. 0123456789
2. 10
3. 012345678910
4. compile time error
5. How many types of loops are there?
6. 4
7. 2
8. 3
9. 1
10. Which of the following statements about the while loop is not true?
11. The while loop is a posttest loop.
12. Testing condition is checked before each iteration.
13. The while loop statement must terminate with a semi-colon.
14. None of them
15. What is the final value of x if initially x has the value 1?

**if (x >= 0)**

**x += 5;**

**else if (x >=5)**

**x += 2;**

1. 8
2. 6
3. 1
4. 4

**Section B (20)**

Q.1. Which of the following while statements are equivalent to the following code?

**x= 4;**

**do**

**{**

**y=x+7;**

**x++;**

**} while(x<9);**

x=4;

while(x<9)

{

y=x+7;

x++;

}

y=x+7;

x++;

x=4;

y=x+7;

x++;

while(x<9)

{

y=x+7;

x++;

}

x=4;

while(x<=9)

{

y=x+7;

x++;

}

Q.2. Change the following C++ code from a while loop to a for loop:

**int x;**

**cin>>x;**

**while(x!=10)**

**{**

**cout<<x<<"\t";**

**cin>>x;**

**}**

Q.3. What is the difference between if-else-if and switch statements?

Q.4. If originally x=1, y=0, and z=1, what is the value of x, y, and z after executing the

following code?

**if(x>y && x>z) {y=x;z=x+1;}**

**else if(x+y>=z) {x++;z=x+1;}**

**else y=z+x;**

Q.5. Write a program in c++ that input a number from user and prints its reverse using a loop.

E.g. if users input 2345, the output should be 5432 and if input is 567891, the output

should be 198765.

**Section C (20)**

Q.1. Write a program to input a number N and prints its table up to 10.

Q.2. Write a program that prints prime N numbers. N is input by the user. i.e. if N is 10,

print first 10 prime numbers.

Q.3. Write a program to output the following pattern using nested loops.


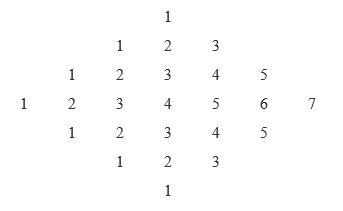


Time: 60 min

**Attempt all the Questions. Each question carries equal points**.

**Question 1** Point out the error, if any in the program.

1. **#include<stdio.h>**
2. int main()
3. {
4. int a = 10, b;
5. a >=5 ? b=100: b=200;
6. printf("%d\n", b);
7. return 0;

**Question 2**  Write a program to print your registration # on screen.

**Question 3**  Write a program to display the following output using a single cout statement.

Subject Marks
Mathematics 90 
Computer 77 
Chemistry 69

**Question 4** What is the output of following program?

int result = 4 + 5 * 6 + 2;
cout << result;

int a = 5 + 7 % 2;
cout << a;

**Question 5** Write a program which accept two numbers and print their sum.

**Question 6** What is the output of following program?

int x = 10, y;
y = x++;
cout << y;

int x = 10, y;
y = x++;
cout << x;

int x = 10;
x++;
cout << x;

int x = 10, y;
y = ++x;
cout << y;

int x = 10;
cout << ++x;

int x = 10;
cout << x++;

**Question 7** Write a program to check whether the given number is positive or negative (using ? : ternary operator )

**Question 8** Write a program that takes length as input in feet and inches. The program should then convert the lengths in centimeters and display it on screen. Assume that the given lengths in feet and inches are integers.

Based on the problem, you need to design an algorithm as follows:
1. Get the length in feet and inches.
2. Convert the length into total inches.
3. Convert total inches into centimeters.
4. Output centimeters.

To calculate the equivalent length in centimeters, you need to multiply the total inches by 2.54. Instead of using the value 2.54 directly in the program, you will declare this value as a named constant. Similarly, to find the total inches, you need to multiply the feet by 12 and add the inches. Instead of using 12 directly in the program, you will also declare this value as a named constant. Using a named constant makes it easier to modify the program later.

To write the complete length conversion program, follow these steps:
1. Begin the program with comments for documentation.
2. Include header files, if any are used in the program.
3. Declare named constants, if any.
4. Write the definition of the function main.

| **Question 9** | If the ages of Ram, Sulabh and Ajay are input by the user, write a program to determine the youngest of the three. |
| --- | --- |
| **Question 10** | Write a program to calculate the monthly telephone bills as per the following rule:  Minimum Rs. 200 for upto 100 calls.  Plus Rs. 0.60 per call for next 50 calls.  Plus Rs. 0.50 per call for next 50 calls.  Plus Rs. 0.40 per call for any call beyond 200 calls. |
| **Question 11** | The marks obtained by a student in 5 different subjects are input by the user. The student gets a division as per the following rules: Percentage above or equal to 60 - First division  Percentage between 50 and 59 - Second division  Percentage between 40 and 49 - Third division  Percentage less than 40 - Fail  Write a program to calculate the division obtained by the student. |
| **Question 12** | Any character is entered by the user; write a program to determine whether the character entered is a capital letter, a small case letter, a digit or a special symbol. The following table shows the range of ASCII values for various characters. |
| **Characters** | ASCII Values |
| **A – Z** | 65 – 90 |
| **a – z** | 97 – 122 |
| **0 – 9** | 48 – 57 |
| **special symbols** | 0 - 47, 58 - 64, 91 - 96, 123 – 127 |

**Time: 60 min**

**Attempt all the Questions. Each question carries equal points**.

1. What is the output of this program?
2. #include <iostream>
3. using namespace std;
4. int main()
5. {
6. int n = 15;
7. for ( ; ;)
8. cout << n;
9. return 0;
10. }
11. Write a program to calculate the sum of first 10 natural number.
12. Write a program to find the factorial value of any number entered through the keyboard.
13. Write a program to enter the numbers till the user wants and at the end it should display the maximum and minimum number entered.

Name: Registration #:

**Section A**

This section contains 20 items. Read carefully and select only one suitable option. Each question carries 1 mark. Erasing, cutting or overwriting is not allowed. Selecting two or more options for a question will get zero marks for that question.

1. Programs are converted into the machine language with the help of?

A An editor

B. Compiler

C. An operating system

D. None of the above

1. C/C++ variable cannot start with?

A. An alphabet

B. A number

C. A special symbol other than underscore

D. Both (B) and (C)

1. A character variable can at a time store?

A. 1 character

B. 8 characters

C. 254 characters

D. None of the above

1. Which of the following is not a character constant?

A. 'Thank You'

B. 'quest videos- IT learning at its best'

C. '23.56e-03'

D. All of the above

1. Which of the following statements is wrong? Consider all the variables are declared.

A. INT=123;

B. Val='A' * 'B';

C. Is=20 * 'T'

D. Count+5=res;

1. What punctuation ends most lines of C/C++ code?

A. . (dot)

B. ; (semi-colon)

C. : (colon)

D. ' (single quote)

1. Which of the following is not a correct variable type?

A. float

B. real

C. int

D. char

1. Which of the following is a correct comment?

A. */ Comments */

B. ** Comment **

C. /* Comment */

D. { Comment }

1. Which of the following cannot be used as variable names?

A. Letters

B. Digits

C. Underscores

D. Spaces

1. Which of the following is not a valid escape sequence?

A.\t

B.\n

C.\w

D) All of the above

1. Which of the following is allowed in a C/C++ Arithmetic Instruction?

A) [ ]

B) { }

C) ( )

D) None of the above

1. If a is an integer variable, a=7/3; will return a value

A) 2.5

B) 3

C) 0

D) 2

1. What is the result of following statement? Assume all variables are declared.

x=y=z=0;

1. x=0, y=Null, z=Null
2. x=0, y=0, z=0
3. x=0, y=1, z=2
4. the statement is incorrect

## Which is the only function all C/C++ programs must contain?

1. start()
2. system()
3. main()
4. printf()

## Which of following is not a valid name for a C/C++ variable?

## Examveda

## Exam veda

## Both A and B

## None of these

1. Which of the following correctly shows the hierarchy of arithmetic operations in C/C++?
2. / + * -
3. *- / +
4. + - / *
5. / * + -
6. In which order do the following gets evaluated

| 1. | Relational |
| --- | --- |
| 2. | Arithmetic |
| 3. | Logical |
| 4. | Assignment |

1. 2134
2. 1234
3. 4321
4. None of above
5. Which of the following is the correct order of evaluation for the below expression?
   **z = x + y * z / 4 % 2 – 1**
6. / % + - =
7. = * / % + -
8. / * % - + =
9. % / - + =

## Find the output of the following program.

## void main() { int i=01289; printf("%d", i); }

## 0289

## 1289

## 713

## Syntax error

## Which of the following is output statement in C?

## printf

## scanf

## cout

## cin

**Section B**

**Q2.**What is the output of following program?

int result = 4 + 5 * 6 + 2;
printf(“%d”, result);

int a = 5 + 7 % 2;
printf(“%d”, a);

**Q3**. Write a program which accept two numbers and print their sum.

**Q4**. What is the output of following program?

1. int x = 10, y;
   y = x++;
   printf(“%d”, y);
2. int x = 10, y;
   y = x++;
   printf(“%d”, x);
3. int x = 10;
   x++;
   printf(“%d”, x);
4. int x = 10, y;
   y = ++x;
   printf(“%d”, y);
5. int x = 10;
   printf(“%d”, ++x);
6. int x = 10;
   printf(“%d”, x++);

**Q5**. Swap the values of two variables.

1. With using third variable.
2. Without using third variable.

**Section C**

**Q6**. If the ages of Nasir, Tariq and Iftikhar are input by the user, write a program to determine

the youngest of the three.

**Q7**.Write a program to calculate the monthly telephone bills as per the following rule:
 Minimum Rs. 200 for upto 100 calls.
 Plus Rs. 0.60 per call for next 50 calls.
 Plus Rs. 0.50 per call for next 50 calls.
 Plus Rs. 0.40 per call for any call beyond 200 calls.

**Q8**. Input a five digit number and display it in reverse order?

Hint: Input number is 12345, the output should be 54321 stored in a single variable.
